# Structural insights into the mechanism of pH-selective substrate specificity of the polysaccharide lyase Smlt1473

**DOI:** 10.1016/j.jbc.2021.101014

**Published:** 2021-08-03

**Authors:** Shubhant Pandey, Pranjal Mahanta, Bryan W. Berger, Rudresh Acharya

**Affiliations:** 1 School of Biological Sciences, National Institute of Science Education and Research, Bhubaneswar, Odisha, India; 2 Homi Bhabha National Institute, Mumbai, Maharashtra, India; 3 Department of Chemical Engineering, University of Virginia, Charlottesville, Virginia, USA

**Keywords:** alginate lyase, polysaccharide, biofilm, *Stenotrophomonas maltophilia*, hyaluronic acid, alginate, celluronic acid, CAZyme, carbohydrate-active enzyme, CpHMD, constant pH molecular dynamics, HA, hyaluronic acid, IMAC, immobilized metal ion affinity chromatography, MC, Monte Carlo, MD, molecular dynamics, mdp, molecular dynamic parameter, NPT, normal pressure temperature, NVT, normal volume temperature, PL, polysaccharide lyase, tc, temperature coupling

## Abstract

Polysaccharide lyases (PLs) are a broad class of microbial enzymes that degrade anionic polysaccharides. Equally broad diversity in their polysaccharide substrates has attracted interest in biotechnological applications such as biomass conversion to value-added chemicals and microbial biofilm removal. Unlike other PLs, Smlt1473 present in the clinically relevant *Stenotrophomonas maltophilia* strain K279a demonstrates a wide range of pH-dependent substrate specificities toward multiple, diverse polysaccharides: hyaluronic acid (pH 5.0), poly-β-D-glucuronic (celluronic) acid (pH 7.0), poly-β-D-mannuronic acid, and poly-α-L-guluronate (pH 9.0). To decode the pH-driven multiple substrate specificities and selectivity in this single enzyme, we present the X-ray structures of Smlt1473 determined at multiple pH values in apo and mannuronate-bound states as well as the tetra-hyaluronate-docked structure. Our results indicate that structural flexibility in the binding site and N-terminal loop coupled with specific substrate stereochemistry facilitates distinct modes of entry for substrates having diverse charge densities and chemical structures. Our structural analyses of wild-type apo structures solved at different pH values (5.0–9.0) and pH-trapped (5.0 and 7.0) catalytically relevant wild-type mannuronate complexes (1) indicate that pH modulates the catalytic microenvironment for guiding structurally and chemically diverse polysaccharide substrates, (2) further establish that molecular-level fluctuation in the enzyme catalytic tunnel is preconfigured, and (3) suggest that pH modulates fluctuations resulting in optimal substrate binding and cleavage. Furthermore, our results provide key insight into how strategies to reengineer both flexible loop and regions distal to the active site could be developed to target new and diverse substrates in a wide range of applications.

Polysaccharide lyases (PLs) (EC 4.2.2.-) are a large, diverse, and expanding class of carbohydrate-active enzymes (CAZymes) classified into 41 families based on sequence similarity (www.cazy.org). Each family shows a particular organization of structural elements ranging from α/α toroid of PL-2, 5, 10; β-helix of PL-1, 3, 6, 9, 16; β-sandwich of PL-4, 22; β-jelly roll of PL-7, 13, 14, 18, 19; β-propeller of PL-11, 21, 24, 25 to mixed α/α toroid and antiparallel β-sandwich of PL-8, 12, 15, 17, 20, 23, 27 (1, 2). Typically, each PL is specific to a type of anionic polysaccharide. The PLs act on a structurally and chemically diverse array of anionic polysaccharides of bacterial, algal, fungal, and mammalian origin. PLs play key roles in diverse processes such as biofilm processing, virulence, and biomass conversion and have been explored for use in biotechnological applications such as value-added chemical production from biomass and biofilm removal from multidrug-resistant pathogens. PLs employ a lytic β-elimination (*syn*/*anti*) reaction mechanism to cleave the glycosidic bonds in anionic polysaccharides (Fig. 1, *C* and *D*) (3, 4). The *syn* or *anti* mode of β-elimination reaction largely depends upon the substrate stereochemistry and enzyme catalytic geometry. In β-elimination, both charge neutralization of the carboxylic acid and a general acid/base for proton abstraction are required for catalysis. The role of the charge neutralizer is played by divalent cations such as Ca^2+^ or Mn^2+^, side chains of Asn or Gln residues, or positively charged residues such as Arg and Lys (1). In *anti*-mode, the proton donor and acceptor are different residues; for example, His acting as a proton acceptor and Tyr as a proton donor. In *syn*-mode, a single residue plays a dual role; for example, Tyr acting as both donor and acceptor for a number of PLs (1).

**Figure 1:**
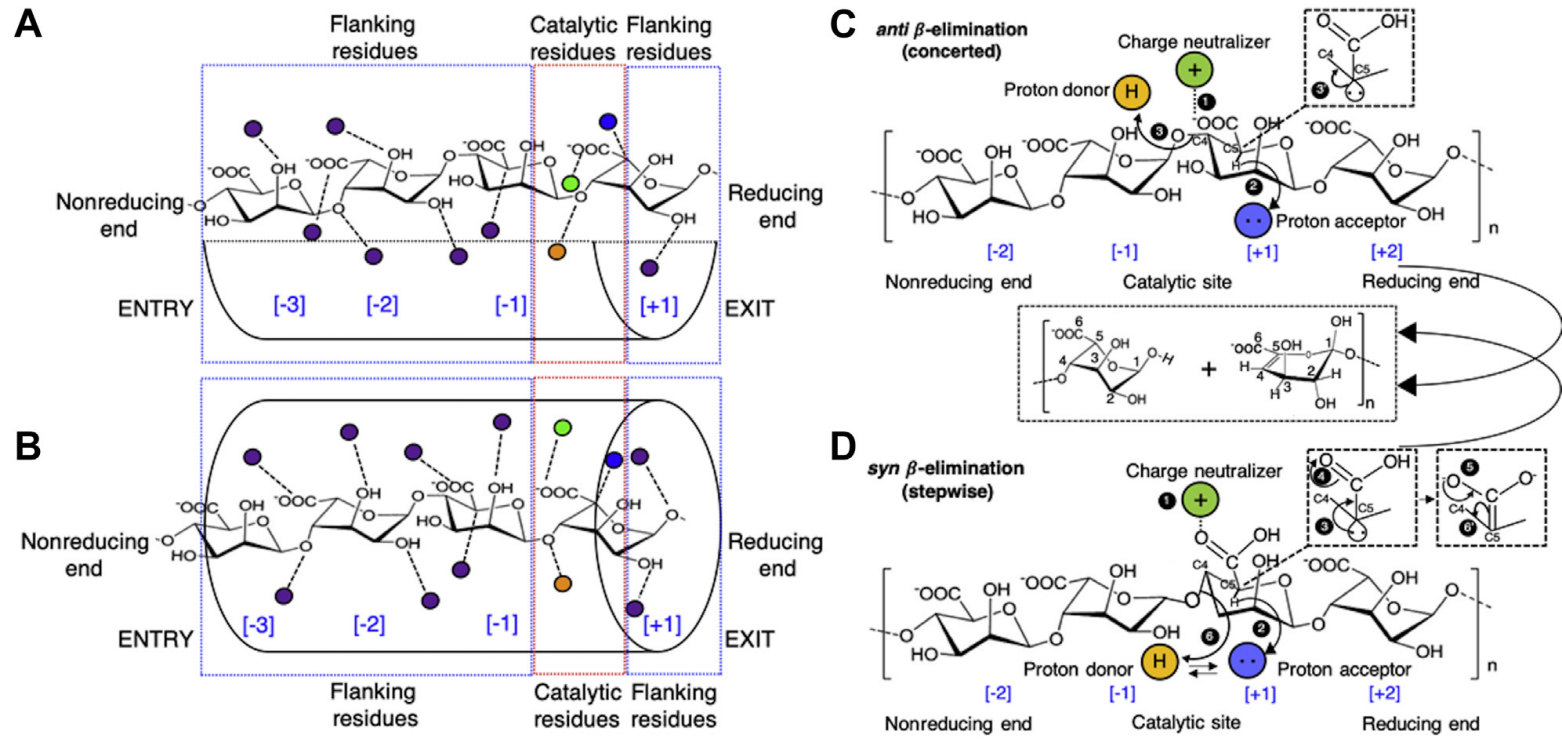
**Schematics of active site architecture among polysaccharide lyases, *syn*/*anti*-mode of *β*-elimination reaction mechanism.** Cleft architecture (*A*), tunnel architecture (*B*). The schematics shows a bound alginate tetramer entered from its reducing end, *purple spheres* enclosed by *blue box* represent flanking residues; *orange*, *blue*, and *green spheres* enclosed by a *red box* represent catalytic residues. The substrate-binding sites, which include the flanking and catalytic residues, are subdivided as numbered subsite [−3, −2, −1, +1], where −1, +1 represents the catalytic site. Proposed *β*-elimination reaction mechanism for PLs (*C* and *D*). Alginate from nonreducing to reducing end has been marked as [−2, −1, +1, +2]. The subsite [−1, +1] corresponds to the catalytic site, and rest of them correspond to substrate-binding site. At catalytic site “Charge neutralizer” is a divalent cation such as Ca^2+^, Mg^2+^, Mn^2+^, or side chain of a polar amino acids such as Asn/Gln, or positively charged residues such as Arg/Lys. Tyr acts as Proton donor (Br∅nsted acid) in almost all of the cases, whereas His generally acts as proton acceptor (Br∅nsted base). Tyr can act as both proton donor and acceptor for particular cases. *anti*-*β*-Elimination reaction mechanism, ^(1)^ Charge neutralization of C5-COO^−^ group lowers the C5-H pKa, ^(2)^ Proton acceptor abstract the C5-H creating a carbanion (shown in *inset*) at C5 position and this leads to a short-lived high-energy transition state that decays by concomitant steps resulting in cleavage of glycosidic bond ^(3)^, and formation of unsaturated bond (C4=C5) at new nonreducing end ^(3’)^ (*C*). *syn-β*-Elimination reaction mechanism, ^(1)^ Charge neutralization of C5-COO^−^ group lowers the C5-H pKa, ^(2)^ Proton acceptor abstract the C5-H and transform into a proton donor, lone pair of electron on C5 ensue the formation of enolate ion intermediate (shown in *inset*) ^(3, 4)^, enolate ions rearrange and lead to concomitant formation of double bond (C4=C5) ^(5, 6’)^ and cleavage of glycosidic bond ^(6)^ (*D*). For *syn*-*β*-elimination reaction to happen, the same residue (*e.g.*, Tyr) has to act simultaneously as proton acceptor and donor. In *syn* mode of reaction both C5-H and glycosidic oxygen have to be in the same plane, while for *anti*-mode this orientation is opposite. The choice of *syn*- or *anti-β*-elimination depends on enzyme catalytic geometry and substrate stereochemistry.

For PLs, the catalytic site can either be a cleft or tunnel, suited for binding anionic polysaccharide chain of variable length and chemical composition. The substrate enters the binding cleft/tunnel through the reducing end (C1-OH). After binding, the reducing end lies at the “exit site,” whereas the nonreducing end (C4-OH) lies at the “entry site” (Fig. 1, *A* and *B*). In between these two extremes lies the “substrate-binding site,” where the rest of the substrates interact with the substrate-binding amino acid residues. These residues can also be referred to as active site flanking residues or simply “flanking residues.” The flanking residues play a significant role in the acquisition, translocation, orientation, and positioning of substrates at the catalytic site located mainly toward the exit site. By convention, the constituting hexose units of the substrates starting from the nonreducing to reducing end are numbered as [−n, −2, −1, +1, +2, +n], with the subsite [−1, +1] corresponding to the catalytic site (Fig. 1). Each subsite within a particular PL has a unique composition of amino acids suited for its site-specific functions. The network of specific enzyme–substrate interactions within a subsite can vary significantly from one subsite to another and is the major determinant of substrate specificity as well as enzyme efficiency. The binding site's intrinsic chemical properties that dictate enzyme–substrate interactions are affected by many extrinsic factors, such as the presence or absence of cations, solution pH, and even the type of interacting substrate. Among these factors, pH plays a vital role in enzyme catalytic activity (5, 6, 7). We previously reported that the PL-5 member Smlt1473 exhibits a pH-dependent, multiple substrate specificity, cleaving both uronic acid– and N-acetyl-glucosamine–containing polymers (5, 6). Among PL families, and even in CAZymes, Smlt1473 is the first such enzyme to possess a pH-directed, multiple substrate specificity. Specifically, Smlt1473 cleaves a glycan (hyaluronic acid; polyHA) at pH 5, poly-β-D-glucuronic acid (polyGlcA) at pH 7, and alginates, poly-β-D-mannuronic acid (polyManA), and poly-α-L-guluronate (polyGulA) at pH 9 (5). Simulations based on homology models of Smlt1473 with docked substrates indicate the importance of a flexible loop region in substrate recognition and turnover (8). However, the specific mechanism by which pH dictates specificity and catalytic activity for a given substrate in a single enzyme has not yet been elucidated.

Smlt1473 is a 36-kDa, secreted enzyme present in strain K279a of *Stenotrophomonas maltophilia*, originally isolated from a bloodstream infection (9, 10); other environmental *S. maltophilia* isolates such as R551–3 do not contain a gene encoding for the PL Smlt1473. *S. maltophilia* strain k279a is a biofilm-producing strain, and prior studies have demonstrated that clinical *S. maltophilia* isolates such as k279a produce branched, complex biofilm exopolysaccharides with varying monomer composition (11, 12). Furthermore, we demonstrated that Smtl1473 is capable of cleaving the mammalian tissue substrate hyaluronic acid (HA), further suggestive of a potential role for Smlt1473 in degrading both microbial polysaccharides during biofilm formation and mammalian tissue polysaccharides during infection. Since Smlt1473 is a secreted enzyme (5), it may be likely that the observed pH-dependent activity allows heterogeneity in biofilm formation or tissue degradation in response to different external environmental conditions. Thus, Smlt1473 represents an important and interesting enzyme to characterize structurally in order to gain further insight into biological roles of PLs in both biofilm formation and possible virulence.

To investigate the structural basis for the unique, pH-driven ability to cleave multiple substrates in a single enzyme, we have overexpressed and purified Smlt1473 wild-type and mutant proteins and crystallized the “apo” and “substrate-bound” forms at a wide range of pH values (5.0, 5.5, 6.5, 7.0, 8.5, and 9.0). Successful crystallization trials over a broad spectrum of acidic to basic pH values reflect this enzyme's robust pH stability and enzyme–substrate complex formation. We have determined the structures of Smlt1473 (wild-type and active site mutant) in the apo, substrate-bound (tetra-ManA, Hexa-ManA), and docked (tetra-HA) forms. Our results show that the catalytic architecture is not an open cleft used in previous computational work (8), but rather a tunnel. Furthermore, it yields insights into the unique access to the substrate-binding site in the catalytic tunnel of Smlt1473. It explains how the interplay of overall enzyme conformational flexibility, substrate configurations, and active site charge density as a function of pH creates a mechanism by which pH-dependent substrate turnover occurs. The strict pH-dependent mechanism of substrate turnover further allowed us to generate arrested Michaels–Menten complexes for wild-type Smlt1473-ManA at catalytically inactive pH 5.0 and 7.0 in order to determine specific molecular interactions responsible for binding *versus* catalysis. Moreover, the pH-trapped substrate-bound structures have catalytical relevance and represent a pretransition binding state. Of interest, although each of the pH optima for specific substrate turnover in Smlt1473 is consistent with a single optimum pH for activity among other PLs, our work further develops the role of pH in modulating the microenvironment for guiding structurally and chemically unique substrate to access the substrate-binding site. Our analyses on the pH-trapped substrate-bound structures suggests that atomic fluctuation in the enzyme catalytic tunnel is preconfigured and pH helps to tune the optimal fluctuation resulting in pH-dependent optimal binding and cleavage.

## Results

### General description of Smlt1473 crystal structures

**Figure 2:**
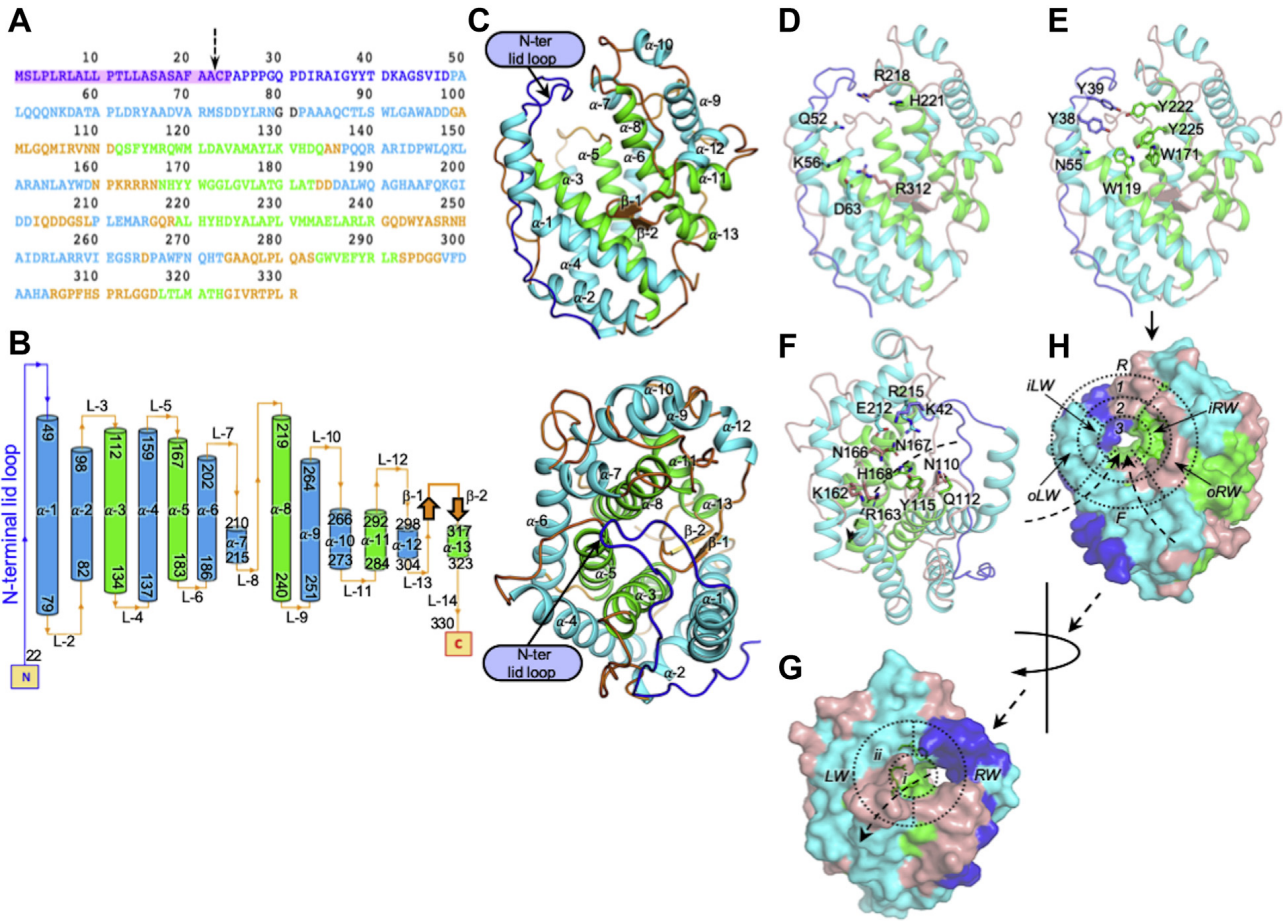
**Protein sequence, two-dimensional topology map, and structural description of Smlt1473 in apo form.***A*, the full-length sequence of Smlt1473 (M1-R331), the sequence highlighted in *magenta* (M1-P24) is lipoprotein signal peptide, the sequence (A22-L330) was obtained after purification and set for crystallization at different pH. *B*, two-dimensional topology map of Smlt1473 showing arrangement and connection between different structural elements from N-ter to C-ter, *sky-blue* and *green cylinders* represent outer and inner *α*-helices, respectively, *orange arrows* represent β-strands, and the interconnecting loops are represented by *orange lines*. *C*, the three-dimensional crystal structures shown in *cartoon form*, entry site view (i), *top view* represents the Smlt1473 architecture, *i.e.*, incomplete (*α*/*α*)_toroid_ fold (ii), inner and outer *α*-helices are colored *green* and *cyan*, respectively, loops and the β-strands are colored *salmon* except N-terminal lid loop, which is colored *blue*. The substrate-binding tunnel of Smlt1473 showing the substrate entry and product exit sites (*D*–*H*). The *dashed arrow* (*G* and *H*) represents path of substrate egress, the *head of the arrow* is pointing toward the exit site and the *tail* represents the entry site. *Cartoon* representation shows entry (*D*), tunnel site (*E*), and exit site (*F*), and *sticks* represent respective constituent amino acids; (*G*) and (*H*) are surface representation of (*F*) and (*D* and *E*), respectively. The nomenclatures (*R*, *iLW*, *oLW*, *F*, *oRW*, *iRW*, *1*, *2*, *3* and, *LW*, *RW*, *i*, *ii*) are detailed in the Results and Discussion sections of the text.

Smlt1473 wild-type and mutants were purified, buffer exchanged, and crystallized at different pH values (acidic to basic) (6). Structures at each pH value were solved to high resolution to resolve specific substrate–enzyme interactions and changes in the enzyme structure. The details of data collection and refinement statistics are summarized in Table 1 and Table S1. The protein used for crystallization has a truncated N terminus (M1-A21) due to cleavage by signal peptidase between A21 and A22 to remove the signal peptide sequence present in Smlt1473 (5) (Fig. 2*A*). We have built and refined the structures from residues A22-L330 (Fig. 2); electron density for other residues outside this range was either weak or absent. The structure is composed of 13 α-helical segments (α-1 to α-13) connected by 14 loops (L-1 to L-14) and two β-strands (β-1 and β-2) (Fig. 2, *B* and *C* (i and ii)). The outer (cyan) and inner (green) rings of α-helical segments are organized into an (α/α)_5_ incomplete toroid fold (Fig. 2*C* (ii)). The N-terminal extended loop (blue) forms an interacting lid that forms the roof of the Smlt1473 substrate-binding tunnel (Fig. 2*C* (i and ii)), referred to as “N-terminal lid loop” (13). Keeping the path of substrate egress in mind, we have divided our structural analysis into two main parts: (i) the substrate entry site (Fig. 2, *D*, *E*, and *H*) and (ii) the product exit site (Fig. 2, *F* and *G*). The surface representations of the entry and exit sites (Fig. 2, *G* and *H*) give a distinct structural view of substrate entry and exit that can be described in terms of raised and concentric circular surfaces. In particular, they portray a possible structural role played by distinct structural elements within Smlt1473 such as the N-terminal lid loop, inner ring of helices, outer ring of helices, and the connecting loops described earlier (Fig. 2*C* (i and ii)). At the entry site, the substrate-interacting surface is defined by helix *α*-1 (P49-N79), N-terminal lid loop (Y38-Y39), L-8 (Q217-R218), *α*-8 (H221-Y222, Y225), L-11(G274-S283), L-13 (R305-D315), β-1 (H309-P311), β-2 (G314-D316), *α*-5 (W171), and *α*-3 (Q112, Y115) (Fig. 2, *D* and *E*).

**Table 1:** Crystallographic data collection and refinement statistics

Proteins	Smlt1473(wt) pH 5.0 (apo)	Smlt1473(wt) pH 7.0 (apo)	Smlt1473(wt) pH 9.0 (apo)	Smlt1473(wt) pH 5.0 (tetra-ManA)	Smlt1473(wt) pH 7.0 (hexa-ManA)	Smlt1473(H168A) pH 5.0 (tetra-HA)
PDB Deposition ID	7FHX	7FHY	7FHZ	7FI0	7FI1	7FI2
Data Collection Statistics						
Radiation source	NISER home source, BRUKER-PROTEUM	NISER home source, BRUKER-PROTEUM	NISER home source, BRUKER-PROTEUM	NISER home source, BRUKER-PROTEUM	NISER home source, BRUKER-PROTEUM	NISER home source, BRUKER-PROTEUM
Wavelength (Å)	1.5418	1.5418	1.5418	1.5418	1.5418	1.5418
Detector	Photon100	Photon100	Photon100	Photon100	Photon100	Photon100
Temperature (K)	100	100	100	100	100	100
Space group	P 2_1_2_1_2_1_	P1	P2_1_2_1_2	C222_1_	P2_1_2_1_2	P3_1_21
Unit Cell Dimensions: a, b, c (Å), ⍺, *β*, *γ* (°)						
a, b, c (Å)	49.131, 94.737, 160.499	43.652, 57.943, 67.569	47.983, 160.579, 46.942	105.757, 160.662, 48.160	48.139, 160.228, 46.878	75.564, 75.564, 112.360
⍺, *β*, *γ* (°)	90.00, 90.00, 90.00	71.14, 89.71, 73.79	90.00, 90.00, 90.00	90.00, 90.00, 90.00	90.00, 90.00, 90.00	90.00, 90.00, 120.00
Resolution (Å)	47.36–2.63 (2.73–2.63)^a^	29.09–2.20 (2.3–2.2)^a^	41.19–2.45 (2.55–2.45)^a^	29.45–2.31 (2.41–2.31)^a^	35.76–2.43 (2.53–2.43)^a^	65.44–2.60 (2.70–2.60)^a^
I/σ(I)	14.36 (2.62)^a^	9.33 (2.94)^a^	10.52 (1.87)^a^	10.04 (2.14)^a^	20.24 (1.99)^a^	16.59 (4.07)^a^
Multiplicity	10.35 (3.86)^a^	5.24 (3.24)^a^	6.94 (1.69)^a^	6.39 (2.88)^a^	12.80 (2.47)^a^	10.07 (4.25)^a^
Completeness (%)	91.2 (71.28)^a^	98.7 (97.0)^a^	97.4 (86.1)^a^	95.8 (80.9)^a^	94.8 (76.7)^a^	96.4 (78.3)^a^
R_merge_ (%)	15.98 (51.77)^a^	14.22 (44.53)^a^	13.68 (38.97)^a^	15.50 (55.97)^a^	10.00 (53.79)^a^	11.40 (33.13)^a^
CC1/2	0.988 (0.635)^a^	0.991 (0.740)^a^	0.971 (0.545)^a^	0.989 (0.578)^a^	0.993 (0.338)^a^	0.996 (0.909)^a^
Total no. of reflections	21,068 (1737)^a^	30,028 (3689)^a^	13,694 (1338)^a^	17,710 (1763)^a^	13,632 (1217)^a^	11,515 (1013)^a^
Data Refinement Statistics						
Resolution (Å)	47.30–2.63	29.05–2.20	35.73–2.50	29.40–2.31	35.72–2.43	65.31–2.60
Number of reflections used in refinement	19,993	28,418	12,968	16,800	12,908	10,936
R_work_/R_free_	0.2246/0.2718	0.2208/0.2576	0.2549/0.2860	0.2049/0.2515	0.2286/0.2669	0.1873/0.2316
ΔR (%)	4.72	3.68	3.11	4.66	3.83	4.43
Solvent content (%)/Matthews coefficient (Å^3^/Da)	54.19/2.71	44.24/2.22	53.26/2.65	55.21/2.77	51.66/2.56	52.90/2.63
No. of Molecule/asu (Z′)	2	2	1	1	1	1
No. of atoms						
Protein	4840	4777	2385	2418	2414	2402
Water and others (ligands or ions)	90	191	50	222	96	111
B-factors						
Wilson B-factor (Å^2^)	13.50	17.60	11.20	18.10	29.10	21.90
Overall B factor (Å^2^)	13.87	26.42	14.73	21.95	34.42	22.08
Proteins (Å^2^)	13.93	26.40	14.69	21.70	34.50	21.95
Ligand/ions (Å^2^)	34.51	31.56	41.63	39.20	41.04	43.79
Waters (Å^2^)	4.13	26.28	10.57	23.09	25.63	17.79
RMSD from ideal values						
Bonds lengths (Å)	0.006	0.009	0.004	0.009	0.007	0.008
Bond angles (^o^)	1.412	1.568	0.782	1.604	1.504	1.568
Ramachandran plot statistics (%)						
Favored	97.9	97.7	98.0	97.7	98.0	96.7
Allowed	2.1	2.3	2.0	2.3	2.0	3.3
Outliers	0	0	0	0	0	0

R_work_=∑||F_o_| − |F_c_||/∑|F_o_|.

R_free_ is the R_work_ value for ∼5% of the reflections excluded from the refinement.

R_merge_ =∑(I − <I>)/∑I.

ΔR% = (R_free_ − R_work_) × 100.

a Values in parentheses are for the highest resolution shell.

Recalling the structural representation used to organize different regions involved in substrate entry and exit, we have divided the surface representation of the substrate entry site by three dashed concentric circles numbered as 1, 2, and 3 (Fig. 2*H*). The space between concentric circles 1 and 2, and 2 and 3 are further subdivided as *R1*, *oLW*, *F1*, *oRW* and *R2*, *iLW*, *F2*, *iRW* respectively. The *α*-1 forms the inner (*iLW*) and outer left walls (*oLW*) and floor (*F1*); the N-terminal lid loop and L-8 form the roof (*R1*, *R2*); *α*-3, 5, β-1, β-2, L-13 form the floor (*F2*); *α*-8 forms the inner right wall (*iRW*); and L-11 forms the outer right wall (*oRW*) of the catalytic tunnel (Fig. 2*H*). The residues constituting *R1* (S45, V46, I47 P49, Q217), *oLW* (Q53, D57, A60, P61), *F1* (R64, Y65, D68, R71, D74), and *oRW* (L278, P279, L280, Q281, R305, G306, P307, F308, H309) maintain the breadth of the entry site, thereby directly affecting the acceptance of structurally different substrates. The residues constituting *R2* (Y39, R218), *iLW* (Y38, Q52, N55, K56), *F2* (D63, R312), and *iRW* (W171, H221, Y222, Y225) form the region containing key residues demonstrated previously to affect enzyme–substrate catalysis (5, 6). The residues Q112, N167, and H168 form the surface of the third and innermost circle, which is the region containing the key catalytic residues (N167, H168, and Y222) necessary for conversion of bound anionic polysaccharides. Rotating the structures shown in Figure 2, *D*, *E*, and *H* by ∼180°, we observe various representations of the product exit site (Fig. 2, *F* and *G*). The product exit site surface is formed predominantly by contributions of the N-terminal lid loop, L-3 (G99-D111) and L-5 (160–166) (Fig. 2, *F* and *G*). A green patch (*α*-3 and *α*-5) of catalytic residues (N167, H168, and Y222) is visible just at the start of the exit site, which indicates that the catalytic site is situated toward the exit of tunnel. The exit site surface can be divided into two rings of interacting surfaces, rings (*i*) and (*ii*) (Fig. 2*G*). Ring (*i*) comprises the residues Y115 (*α*-3), R215 (*α*-7), and N166 (L-5), and ring (*ii*) comprises K42 (N-terminal lid loop), K162, R163, (L-5), and N109, N110, D111 (L-3); D111 is not shown in the stick representation of Figure 2*F* for sake of clarity. Furthermore, as denoted in Figure 2*G*, both rings are divided into halves: *LW* (left wall) and *RW* (right wall). These surface nomenclatures have been used in the subsequent result sections where we have analyzed the Smlt1473’s catalytic surface.

### Cocrystallized ManA (pH-trapped) and docked HA Smlt1473 structures [E_wt_S] represent pretransition binding state and explains the catalytic mechanism

**Figure 3:**
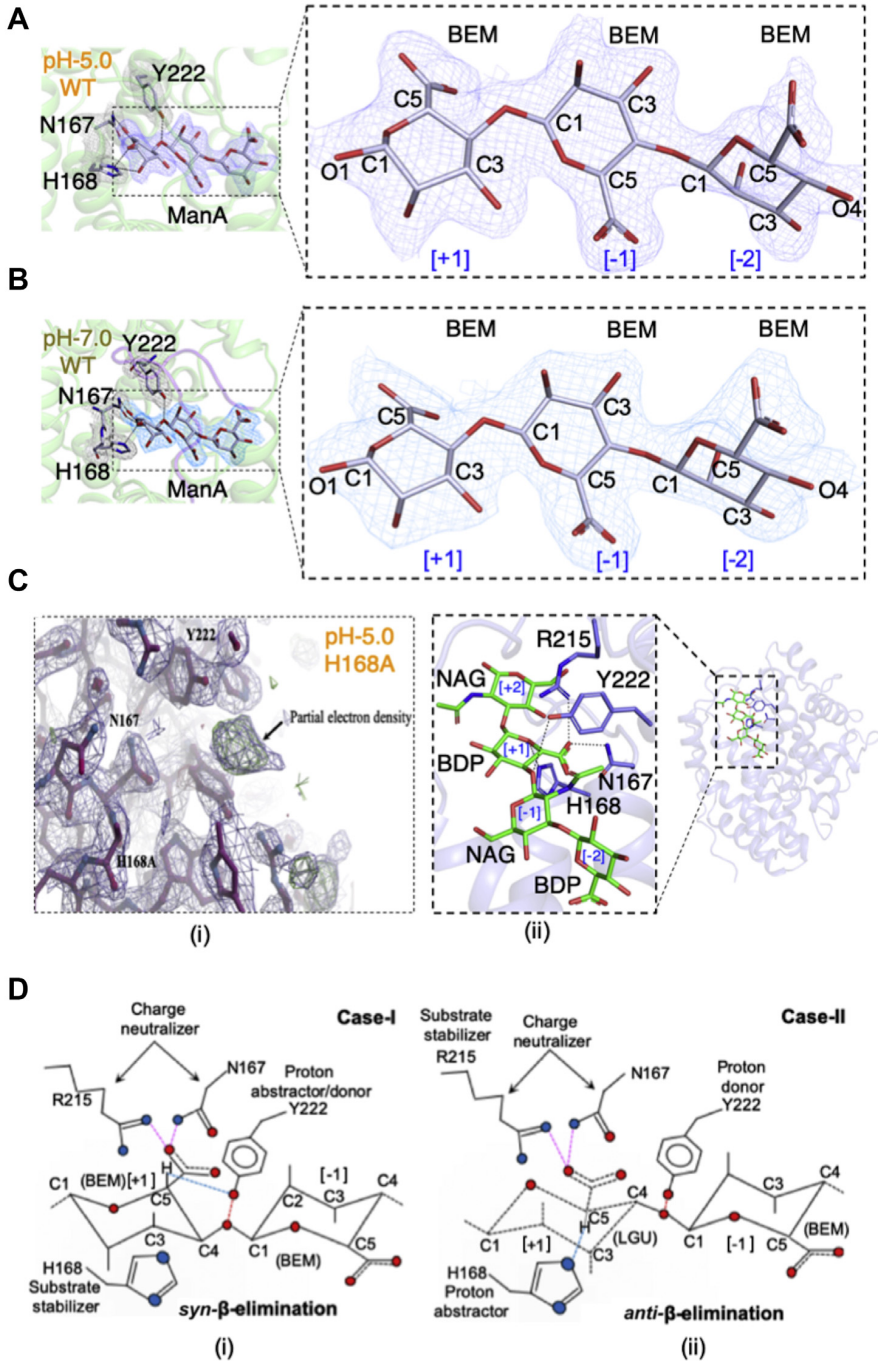
**Substrate-bound structures and catalytic geometry of Smlt1473.** Smlt1473 wt at pH5 (*A*), and pH7 (*B*) cocrystallized with tetra-mannuronic [BEM]_4_ and hexa-mannuronic [BEM]_6_ acid, respectively; electron density maps (2F_O_-F_C_) for substrate and active site residues are contoured at 1.0 sigma level. For both pH 5.0 and 7.0 ManA-complexed structure, only three sugar rings (real-space correlation coefficient [RSCC] > 0.8) were built. Electron density map of Smlt1473 H168A soaked with tetra-HA (2F_O_-F_C_ contoured at 1.5 sigma level and F_O_-F_C_ contoured at 3.1 sigma level) at active site [*C* (i)]. Tetra-HA-docked wt structure [*C* (ii)] (see Fig. S3 for details). In Case-I [*D* (i)], R215 helps N167 to neutralize the charge of C5-COO^−^ group at the +1 subsite of bound ManA, it allows Y222 (proton abstractor/catalytic base) to abstract the C5-H, which generates a lone pair at C5 position. This led the substrate to enter a high-energy transition state, where subsequently the glycosidic oxygen between +1, −1 subsite accepts a proton from Y222 (proton donor/catalytic acid) and lone pair at C5 becomes bond pair between C5 and C4. As a result, concomitant cleavage of glycosidic bond and double bond formation at new nonreducing end occurs. The Case-II [*D* (ii)] explains that Y222 will act as catalytic acid, whereas H168 can act as base in two conditions: if C5-H goes below the plane during any intermediate transition state or if the incoming substrate at the +1 subsite has C5-H below the plane (*e.g.*, GulA (LGU) or IdoA, which is C5 epimer of ManA and GlcA, respectively).

We took advantage of the strict pH dependence for catalytic turnover of substrate by Smlt1473 to trap intact substrate at a pH where turnover does not occur in order to gain further insight into substrate binding. In particular, we cocrystallized tetra-ManA and hexa-ManA, at pH 5.0 and 7.0, respectively; note that maximal specific activity for poly-ManA is at pH 9.0. As anticipated, we locked the ManA substrate into a bound state within the wild-type Smlt1473 crystal structure at both pH 5.0 and 7.0 (Fig. 3, *A* and *B*). To our knowledge, this is the first crystal structure reported for a PL where pH was used to trap the substrate in a bound form within an inactive wild-type structure; other PLs of known structure (Table S2) describe a specific substrate-enzyme pairing at a given pH value, rather than multiple substrate-enzyme pairings at differing pH values. However, in both pH 5.0 and pH 7.0 cocrystal structures, we were able to model only three sugar units of ManA at [+1, −1, −2] subsites. For pH 5.0, we could have modeled the fourth sugar unit (−3 subsite), but we did not owing to real-space correlation coefficient <0.80 (∼0.72, data not shown). For pH 7.0, at other subsites (−3 to −5) the electron density map (2F_O_-F_C_) at 1.2 sigma level was not clear enough for appropriate structural modeling. The loss of electron density at these subsites can be attributed to the fact that, at any given time, the catalytic tunnel can stabilize the interaction of only three units within a polyuronic acid. In addition, we attempted to cocrystallize the tetra-HA substrate complexed with inactivating H168A mutant at pH 5.0, which is the optimum pH for HA catalysis. In the case of the tetra-HA cocrystallized structure, we observed diffuse electron density that was too large for water or any other small molecule present during crystallization (Fig. 3*C* (i)). However, we were unable to resolve an individual sugar unit within the region of diffuse electron density, as it was not large enough to accommodate a pyranose ring including its substituents. Thus, to obtain an HA-bound structure we utilized the Rosetta flexible docking protocol; details are provided in the Experimental procedures section (Fig. 3*C* (ii) and Fig. S3). Based on the catalytic site geometry, there are two possible modes of catalysis, either *syn* (Case I) or *anti* (Case II) β-elimination reaction (Fig. 3*D*); further mechanistic details are summarized in Figure 1, *C* and *D* as well. The crystal structure and docking model of substrate-bound structure fulfils the condition for case I. However, in presence of epimers such as GulA (α-L-guluronic acid, C5 epimer of ManA) or IdoA (α-L-iduronic acid, C5 epimer of GlcA) at +1 subsite, case II is possible as C5-H will be below the catalytic plane. Previous work has confirmed enzymatic activity on anionic sugars containing these residues at +1 subsite, but the catalytic magnitude is low compared with ManA- or GlcA-containing sugars (5). Thus, the trapped wild-type [E_wt_S] complex structures will serve as a key starting point for downstream design of active site modifications to further tune substrate specificity and turnover rates.

### Analysis of ManA-complexed Smlt1473 crystal structures as a function of pH

**Figure 4:**
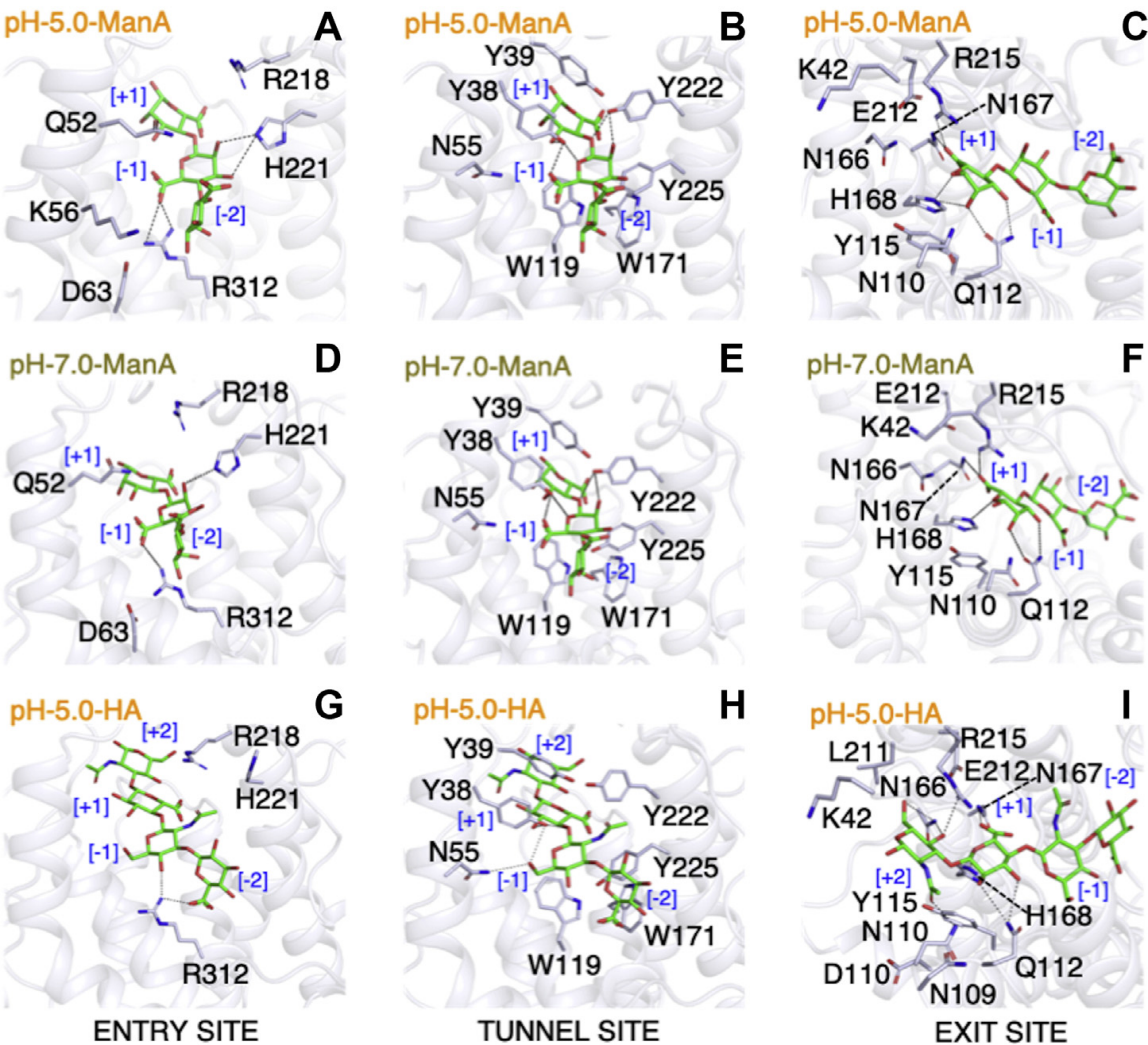
**Enzyme–substrate interactions in Smlt1473 wt structures cocrystallized with ManA (trapped at pH5.0 and 7.0) and docked with HA.** ManA [BEM601_(+1)_ (4←1) BEM701_(−1)_ (4←1) BEM801_(−2)_] complexed structure of Smlt1473 wt trapped at pH5 (*A*–*C*). ManA [BEM401_(+1)_ (4←1) BEM501_(−1)_ (4←1) BEM601_(−2)_] complexed structures of Smlt1473 wt trapped at pH7 (*D*–*F*). Tetra-HA [NAG _(+2)_ (3←1) BDP _(+1)_ (4←1) NAG _(−1)_ (3←1) BDP _(−2)_] docked structures of Smlt1473 wt pH 5 (*G*–*I*). Entry site interacting residues (*A*, *D* and *G*), Tunnel site interacting residues (*B*, *E* and *H*), Exit site interacting residues (*C*, *F* and *I*).

We analyzed the interactions of ManA bound structures determined at pH 5.0 and pH 7.0 and divided the interaction as per the nomenclature of entry, tunnel, and exit site residues (Fig. 4, *A*–*F*, Tables 2 and 3). For the pH 5.0 cocrystallized structure, at the entry site the residues that lie in 5.0-Å vicinity of the substrates are Q52, K56, D63, R312, H221, and R218 (Fig. 4*A*). The entry site residues are placed in such a way that their interaction will be minimal at [−3, −2] subsites and maximal at the [−1] subsite (*e.g.*, R312) (Table 2). Furthermore, this substrate position and interactions serve two useful purposes; first, it enables more facile entry of substrates into the catalytic tunnel by avoiding crowding of interacting residues at the entry site and second, the addition of an extra, residue-specific interaction apart from tunnel site residues stabilizes the catalytically feasible substrate orientation at [−1] subsite. At the tunnel site (Fig. 4*B*), the residues in the vicinity of the substrate are Y38, Y39, N55, W119, W171, Y222, and Y225. Of these residues, only Y38 and Y222 form H-bond interactions with the substrate at [−1, +1] subsite. Y38 forms three interactions of 3.0, 2.8, and 3.0 Å with O5, O6A of BEM701, and O3 of BEM601, respectively (Table 2). Y222, which is the catalytic proton donor/acceptor within the active site, forms two H-bonded interactions of 2.9 and 2.7 Å, respectively, each with O2 of BEM701 and the O4 of the cleavable glycosidic bond between BEM701 and BEM601, consistent with its role in catalysis (Table 2). Finally, at the exit site the residues in the vicinity of the substrate are K42, N110, Q112, Y115, K162, R163, N167, H168, E212, and R215 (Fig. 4*C*). Among these, R215 with its NH1 group forms a salt-bridge interaction of 2.9 Å with O6B of BEM601 (Table 2), thus complementing N167 in neutralizing the carboxylic group. H168 and Q112 form a bifurcated and double H-bond, respectively, with BEM601. Apart from charge neutralization, these interactions are major stabilizers of the sugar ring orientation at the [+1] subsite. The substrate interaction details for hexa-ManA cocrystallized structure are shown in Figure 4, *D*–*F* and Table 3. Comparatively, the pH 5.0 cocrystallized ManA structure has more (11 H-bonds, three salt bridges) and shorter noncovalent interactions than pH 7.0 cocrystallized ManA structure (ten H-bonds, two salt bridges) at the [−1, +1] subsite. This implies an overall low strength of substrate binding at pH 7.0 and thus provides additional evidence beyond the minimal size of the catalytic tunnel's ability to accommodate a trisaccharide as to why we are able to resolve only three sugar units from the cocrystallized hexa-ManA structure.

**Table 2:** Enzyme–substrate interaction for tetra-ManA-complexed Smlt1473(wt) at pH 5

Donor amino acid residue:atom	Residue's position in catalytic tunnel	Acceptor substrate residue:atom	Substrate subsite	Type of interaction	Interaction distance (Å)
H221:NE2	ENTRY SITE (*iRW2*)	BEM701:O2	[−1]	H-BOND	2.9
R312:NH1	ENTRY SITE (*F1*)	BEM701:O6B	[−1]	SALT BRIDGE	2.6
R312:NH2	ENTRY SITE (*F1*)	BEM701:O6B	[−1]	SALT BRIDGE	3.5
Y38:OH	TUNNEL SITE (*R2*)	BEM701:O5	[−1]	H-BOND	3.0
Y38:OH	TUNNEL SITE (*R2*)	BEM701:O6A	[−1]	H-BOND	2.8
Y38:OH	TUNNEL SITE (*R2*)	BEM601:O3	[−1]	H-BOND	3.0
Y222:OH	TUNNEL SITE (*iRW2*)	BEM701:O2	[−1]	H-BOND	2.8
Y222:OH	TUNNEL SITE (*iRW2*)	BEM701:O4:BEM601	[−1, +1]	H-BOND	2.6
Q112:OE1	EXIT SITE (*iiRW*)	BEM601:O2	[+1]	H-BOND	2.8
Q112:NE2	EXIT SITE (*iiRW*)	BEM601:O3	[+1]	H-BOND	2.9
N167:ND2	EXIT SITE (*iLW*)	BEM601:O6B	[+1]	H-BOND	2.9
H168:NE2	EXIT SITE (*iLW*)	BEM601:O2	[+1]	H-BOND	3.4
H168:NE2	EXIT SITE (*iLW*)	BEM601:O5	[+1]	H-BOND	3.0
R215:NH1	EXIT SITE (*iLW*)	BEM601:O6B	[+1]	SALT BRIDGE	2.9

**Table 3:** Enzyme–substrate interaction for hexa-ManA-complexed Smlt1473(wt) at pH 7

Donor amino acid residue:atom	Residue's position in catalytic tunnel	Acceptor substrate residue:atom	Substrate subsite	Type of interaction	Interaction distance (Å)
H221:NE2	ENTRY SITE (iRW2)	BEM501:O2	[−1]	H-BOND	3.1
R312:NH1	ENTRY SITE (F1)	BEM501:O6B	[−1]	SALT BRIDGE	2.7
Y38:OH	TUNNEL SITE (R2)	BEM501:O6A	[−1]	H-BOND	2.6
Y38:OH	TUNNEL SITE (R 2)	BEM501:O5	[−1]	H-BOND	3.3
Y38:OH	TUNNEL SITE (R2)	BEM401:O3	[+1]	H-BOND	3.2
Y222:OH	TUNNEL SITE (iRW2)	BEM501:O2	[−1]	H-BOND	2.9
Y222:OH	TUNNEL SITE (iRW 2)	BEM501:O4:BEM401	[−1, +1]	H-BOND	2.7
Q112:OE1	EXIT SITE (iiRW)	BEM401:O2	[+1]	H-BOND	2.9
Q112:NE2	EXIT SITE (iiRW)	BEM401:O3	[+1]	H-BOND	2.9
N167:ND2	EXIT SITE (iLW)	BEM401:O6A	[+1]	H-BOND	3.0
H168:NE2	EXIT SITE (iLW)	BEM401:O5	[+1]	H-BOND	3.0
R215:NH1	EXIT SITE (iLW)	BEM401:O6A	[+1]	SALT BRIDGE	2.9

### Analysis of tetra-HA-docked Smlt1473 structures

We used the tetra unit of HA [NAG _(+2)_ (3←1) BDP _(+1)_ (4←1) NAG _(−1)_ (3←1) BDP _(−2)_] for cocrystallization with inactive mutant H168A at pH 5.0. After solving the structure, we found a significant region of diffuse electron density within the active site, but we did not observe any specific, resolvable interactions with water or other solutes. Furthermore, we were unable to model an individual sugar unit within the region of diffuse electron density, as it was not large enough to accommodate a pyranose ring including its substituents. Since we know the specific region within the tunnel that corresponds to polysaccharide binding from our high-resolution ManA-complexed enzyme structures, we performed docking studies for tetra unit of HA by taking the protein coordinates of wild-type Smlt1473 bound to tetra-ManA at pH 5. Specifically, we used the Rosetta flexible ligand protocol to generate a large number of possible models, and by utilizing scoring parameters we were able to find the most plausible tetra-HA-docked structure (Fig. S3, *A*–*D*). We analyzed the substrate interaction of docked HA structure in accordance with previous nomenclature (Fig. 4, *G*–*I* and Table 4). At the entry site, considering the residues interacting with tetra-ManA (Q52, K56, D63, R312, H221, R218), for tetra-HA only R312, H221, and R218 are present in the vicinity of the tetra-HA substrate (Fig. 4*G*). R312 has formed a bifurcated interaction of 2.7 and 2.9 Å with O23-BDP (salt bridge) at the [−2] and O14-NAG (H-bond) [−1] subsites, respectively (Table 4). Entering into the tunnel (Fig. 4*H*), the residues encountered are the same as the tetra-ManA bound structure; they are Y38, Y39, N55, W119, W171, Y222, and Y225, of which Y38 and N55 only form H-bond interaction. Y38 forms four interactions of 3.4, 2.8, 3.3, and 2.8 Å with O16-NAG _[−1]_, O15- NAG _[−1]_, NAG _[−1]_-O9-BDP _[+1]_, and O8-BDP _[+1]_ respectively, whereas the N55 residue forms a single H-bond interaction with O16-NAG _[−1]_ (Table 4). The maximum number of interactions are formed at the exit site, in contrast to exit site residues found in tetra-ManA bound structure (K42, N110, Q112, Y115, K162, R163, N167, H168, E212, and R215). We found additional residues in the vicinity of the substrate, such as N109, D111, N166, and L211 (Fig. 4*I*). N110 forms a single H-bond of 2.5 Å with O6 NAG _[+2]_, Q112 forms a bifurcated H-bond of 3.3 and 3.5 Å with O8-BDP _[+1]_ and O7-BDP _[+1]_, respectively (Table 4). N166 forms a bifurcated H-bond of 3.6 and 3.1 Å with O5-NAG _[+2]_ and O3-NAG _[+2]_ , respectively, whereas similar to the tetra-ManA bound structure N167 neutralizes the carboxylate group by forming H-bond of 2.7 Å with O12-BDP _[+1]_ (Table 4). H168 forms a single H-bond interaction of 2.9 Å with O10-BDP _[+1]_, and R215; similar to the tetra-ManA bound structure, this interaction not only helps N167 in neutralization of the carboxylic group but also forms a bifurcated H-bond of 3.0 and 2.9 Å with O12-BDP _[+1]_ and O3-NAG _[+2]_, respectively (Table 4).

**Table 4:** Enzyme–substrate interaction for tetra-HA-docked Smlt1473(wt) at pH 5

Donor amino acid residue:atom	Residue's position in catalytic tunnel	Acceptor substrate residue:atom	Substrate subsite	Type of interaction	Interaction distance (Å)
R312:NH1	ENTRY SITE (*F1*)	BDP:O23	[−2]	SALT BRIDGE	2.7
R312:NH1	ENTRY SITE (*F1*)	NAG:O14	[−1]	H-BOND	2.9
Y38:OH	TUNNEL SITE (*R2*)	NAG:O16	[−1]	H-BOND	3.4
Y38:OH	TUNNEL SITE (*R2*)	NAG:O15	[−1]	H-BOND	2.8
Y38:OH	TUNNEL SITE (*R2*)	NAG:O9:BDP	[−1, +1]	H-BOND	3.3
Y38:OH	TUNNEL SITE (*R2*)	BDP:O8	[+1]	H-BOND	2.8
N55:ND2	TUNNEL SITE (*iLW*)	NAG:O16	[−1]	H-BOND	3.4
N110:ND2	EXIT SITE (*iiRW*)	NAG:O6	[+2]	H-BOND	2.5
Q112:NE2	EXIT SITE (*iiRW*)	BDP:O8	[+1]	H-BOND	3.3
Q112:NE2	EXIT SITE (*iiRW*)	BDP:O7	[+1]	H-BOND	3.5
N166:ND2	EXIT SITE (*iLW*)	NAG:O5	[+2]	H-BOND	3.6
N166:ND2	EXIT SITE (*iLW*)	NAG:O3	[+2]	H-BOND	3.1
N167:ND2	EXIT SITE (*iLW*)	BDP:O12	[+1]	H-BOND	2.7
H168:NE2	EXIT SITE (*iLW*)	BDP:O10	[+1]	H-BOND	2.9
R215:NH2	EXIT SITE (*iLW*)	BDP:O12	[+1]	SALT BRIDGE	3.0
R215:NH2	EXIT SITE (*iLW*)	NAG:O3	[+2]	H-BOND	2.9

### Different modes of substrate binding explain enhancement and reduction of substrate-specific activity upon mutations in the catalytic tunnel

We analyzed the implication of ring puckering on different modes of ManA and HA binding within the catalytic tunnel. Polysaccharides such as HA, which contain both uronic acid and N-acetylglucosamine moieties, can adopt unique secondary structures, including rod, twisted rod, or helical conformations (14, 15, 16, 17). The nature of constituting units, torsion angles, type of linkages, and intramolecular H-bonding are the major contributing factors for adopting a specific secondary conformation. The PLs acting on these polyuronides have adapted structurally to recognize these secondary structure elements for substrate binding and catalysis. There is an interesting periodic display of uronic acid ring face with alternating α- or β-face (Fig. S4) defined by puckering of the ring. It has been observed that binding of polyuronides by enzymes induce a subtle change in the secondary conformation of the polyuronide due to the formation of new intermolecular H-bonds and breakage of old intramolecular H-bonds. However, except for the dihedral angles, binding still does not produce any overall structural change and the uronic acid moieties retain their overall native conformations and face. Thus, the specific conformational features of the substrate are recognized by a given PL to orient the catalytic domain to select the appropriate substrate stereochemistry. For example, in the crystal structure of enzyme-free, sodium-complexed HA, one HA [BDP (1→3) NAG] unit always displays the same face (either **α** or **β**), but the face of the subsequent (1→4) linked BDP unit changes from the preceding HA unit (16). This give rise to the alternate display of **α** and **β** faces, and this conformational pattern is preserved even in the crystal structures of HA-complexed PL-8 HA lyase from *Streptococcus pneumoniae* (18). In these structures, the HA lyase is complexed with “one HA” [**BDP**_−**2**_ (1→3) **NAG**_−**1**_], “two HA” [**BDP**_−**2**_ (1→3) **NAG**_−**1**_ (1→4) BDP (1→3) NAG], and “three HA” units [BDP (1→3) NAG (1→4) **BDP**_−**2**_ (1→3) **NAG**_−**1**_ (1→4) BDP (1→3) NAG] (18, 19, 20). The highlighted residue (in bold) adopts the same conformation in all crystal structures, thus making the interactions at subsite [−2, −1] specific for selecting the β-face of the interacting HA unit, regardless of chain length (Fig. S4).

**Figure 5:**
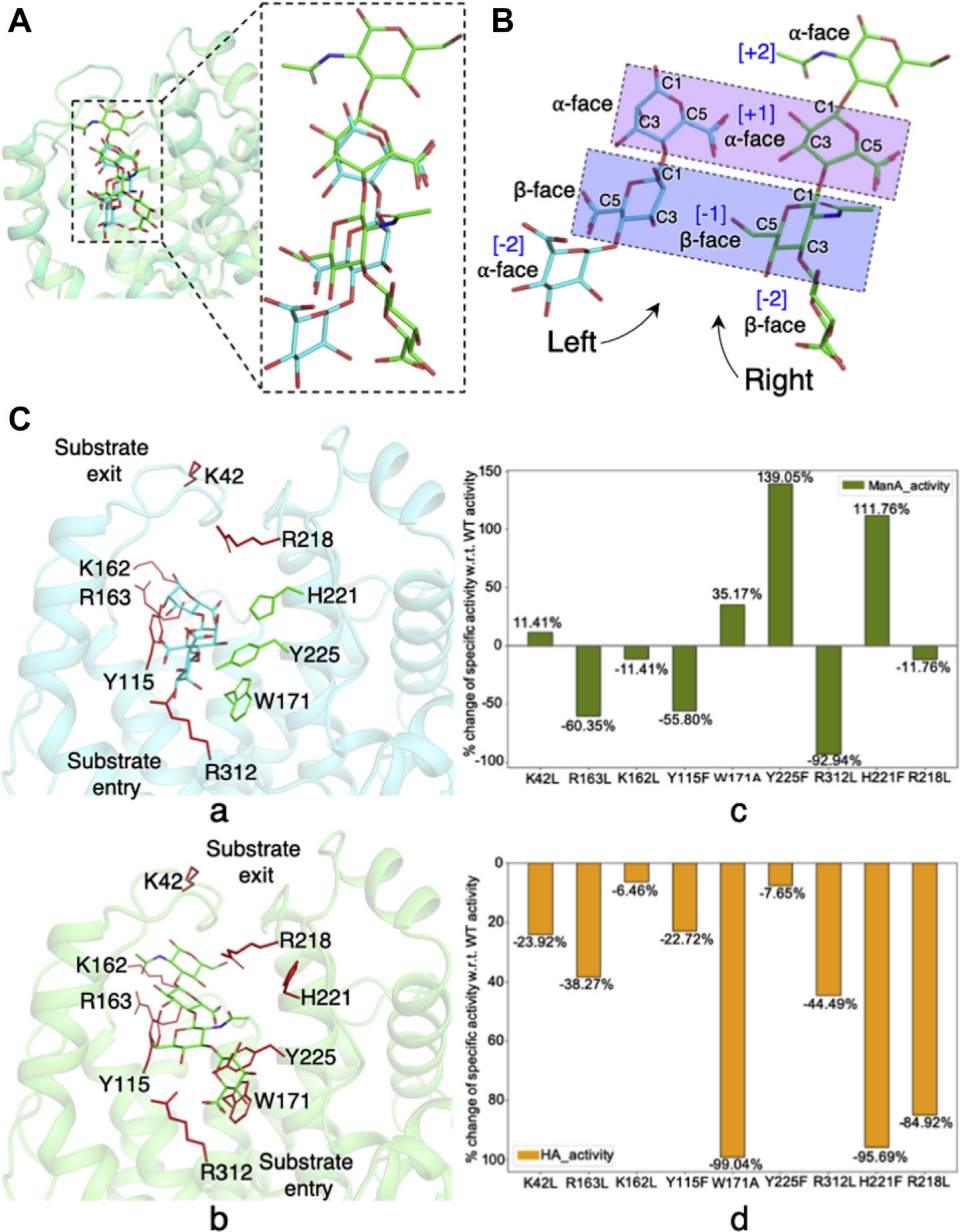
**Structural superimposition of tetra-ManA-complexed and tetra-HA-docked structures showing different modes of substrate entry, binding, and catalytic effect of mutations in path of substrate egress.** The sugar ring puckering pattern (*α*-face [C1, C3, C5 pointing above sugar plane], β-face [C1, C3, C5 pointing below sugar plane]) at catalytic site (+1, −1) is conserved for both substrates; it is different in case of other subsites, which explains the mode of substrate entry is opposite at the entry site (*A* and *B*). This observation is further confirmed by the earlier results (6) of specific enzyme activity of mutated substrate-binding amino acids toward poly-ManA [*C* (a and c)] and poly-HA [*C* (b and d)]. *Red* and *green* colored residues are those whose mutation has negative and positive effect, respectively, on enzyme activity. The bar plots represent the respective substrate-specific percent change in wild-type activity for each mutation for specific substrates.

Given the similar, periodic conformational patterns observed for other polyuronides, it is reasonable to expect that a similar substrate-PL specificity will occur for other PLs and substrates as well. Given that Smlt1473 has demonstrated activity against multiple substrates, it is likely that adopting different modes of substrate binding is necessary to compensate for the conformational variability between substrates and specific activity will correlate with optimal stereochemical configuration of substrate. To analyze the role of puckering on different modes of substrate binding, we superposed the ManA- and HA-bound structures and found that these substrates have different modes of substrate entry; ManA enters from the left side of the tunnel, whereas HA enters from the right side (Fig. 5*A*). We compared the sugar puckering at the catalytic site [+1, −1] and found that puckering for both the substrate is conserved; it is ⍺ (C1, C3, and C5 above the plane) at subsite [+1], which is shown as purple-shaded rectangle in Figure 5*B*, and β (*i.e.*, C1, C3, and C5 below the plane) at subsite [−1], which is shown as blue-shaded rectangle in Figure 5*B*. At the [−2] subsite it is ⍺ for ManA and β for HA, which creates a significant stereochemical twist in the substrate chain; ManA twists toward the left-hand side, whereas the HA twists toward the right-hand side. This difference in orientation at the [−2] subsite also affects the substrate-interacting residues and is a major determinant for the different modes of binding for these substrates (Fig. 5*B*). The left *versus* right positioning observed within the active site is also consistent with prior enzyme kinetic studies, in which for the poly-ManA substrate, mutations on the left side of the tunnel (Y115F, R312L) are detrimental for its specific activity (6). Of interest, mutations to residues on the right side of the tunnel (H221F, W171A, and Y225F) increased the specific activity of poly-ManA, thus providing evidence in favor of a substrate conformational recognition within the tunnel region [Fig. 5*C* (a and c)]. Similarly, for poly-HA all mutations in the tunnel have a negative effect on activity owing to its weaker binding *versus* poly-ManA, but the mutations that are most detrimental to specific activity are present on the right side of the tunnel (W171A, H221F, R218L), again consistent with the observed mode of entry from the HA-bound structure (Fig. 5*C* (b and d)) (6). To validate the stability of binding pose of tetra-HA in docked structure, we performed molecular dynamics (MD) simulations of 100 ns for the tetra-HA-docked Smlt1473 structure. (See Experimental procedures, Figs. S5 and S6, and Note S1).

### B-factor (temperature factor) analysis of crystal structure confirm the relation between pH-directed preconfigured atomic fluctuations and substrate-specific turnover

In order to visualize and understand the effects of pH on Smlt1473 structure, we have crystallized and determined the protein structure at different pH values (5.0, 5.5, 6.5, 7.0, 8.5, and 9.0). The atomic superposition of all the crystal structures across the entire range of pH values did not show any significant change as a function of pH (Fig. S1). However, significant rotameric transitions of important substrate-binding residues such as K42, K162, R163, H221, and R312L have been observed at different pH values (Fig. S2). We have compared all such rotameric transitions with respect to the pH 5.0 structure and the defined entry and exit sites as described previously. At the entry site, the residues involved in rotameric transition are H221 and R312, of which H221 has an altered orientation in all structures above pH 5.0, whereas R312 has an altered orientation only in the pH 7.0 structure due to a hydrogen bonding interaction with Y225. However, at the catalytic tunnel site, there are no such rotameric transitions. At the exit site, K42 exhibits an altered orientation only in the pH 6.5 and 9.0 structures, whereas in pH 5.0 and pH 5.5 there is no change in orientation; interestingly, at pH 7.0 and 8.5 a lack of electron density precluded analysis of the residue orientation. For K162, the altered orientation was observed only for the pH 6.5 and 9.0 structures; for pH 7.0 and pH 8.5 a lack of electron density precluded analysis of residue orientation. For R163, pH 6.5, 7.0, and 9.0 structures have similar rotamers, in contrast to the pH 5.0 and 5.5 structures. The observed rotameric transitions lead us to analyze if these transitions arise owing to change in flexibility of the protein backbone structure as a function of pH.

### B-factor analysis of apo structures at pH 5.0, 7.0, and 9.0

**Figure 6:**
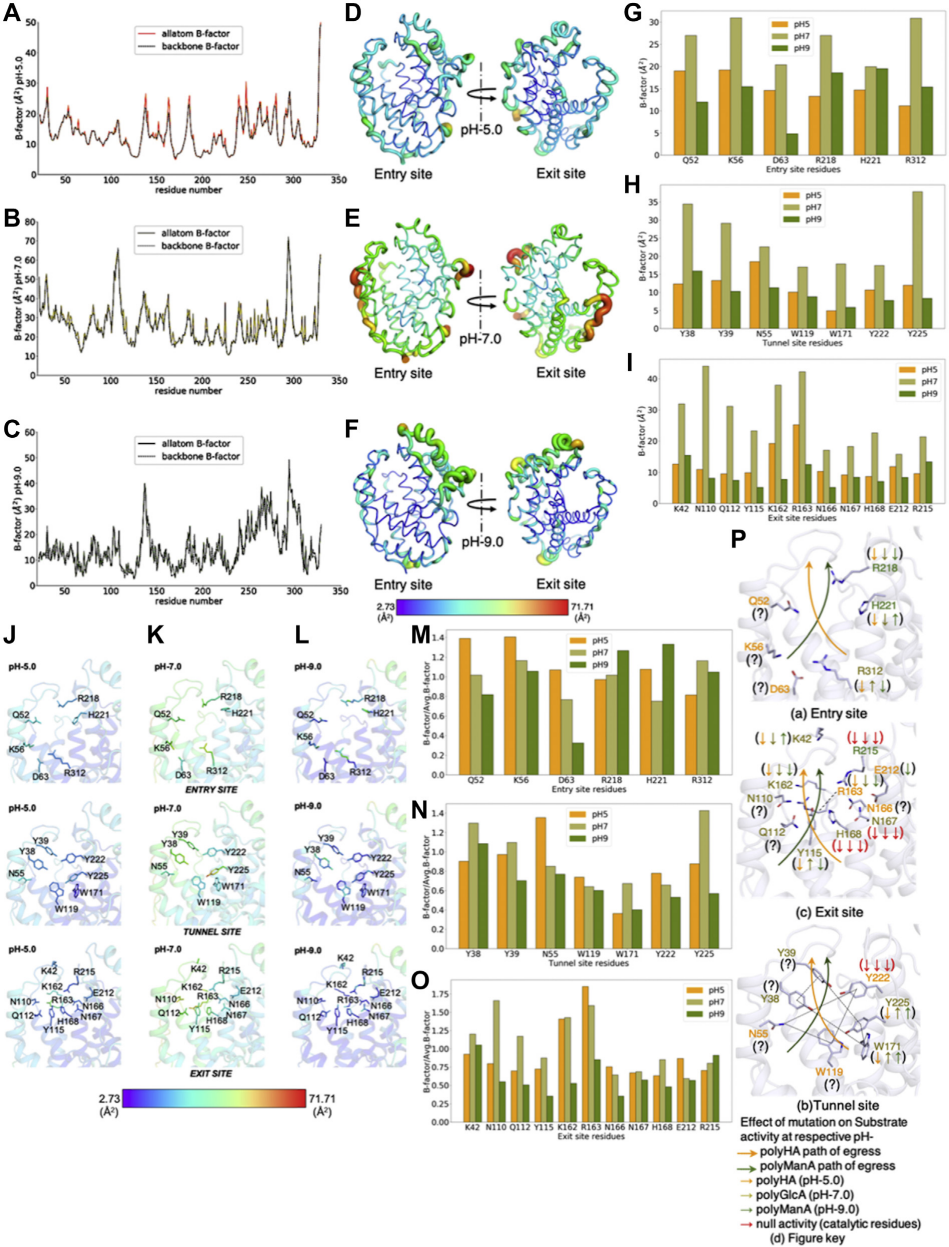
**B-factor plots of Smlt1473 structures solved at different pH.** Plots showing all atom B-factor superposed with backbone B-factor, pH 5.0 (*A*), pH 7.0 (*B*), pH 9.0 (*C*). *Cartoon* representation of Smlt1473 apo structures shown as B-factor putty (*D*–*F*). The B-factor spectrum bar explains colored depiction of the B-factor distribution; *violet* and *red* represent the lowest and the highest value of B-factor, respectively. B-factor plot of entry (*G*), tunnel (*H*), and exit site (*I*) residues. B-factor distribution of catalytic tunnel residues as a function of pH (*J*–*L*). B-factor spectrum colored stick model of residues lining entry, tunnel, and exit site residues at pH 5.0 (*J*), 7.0 (*K*), and 9.0 (*L*) structures. *M*–*O*, represent normalized B-factor values for (*G*–*I*), respectively. *Cartoon* and *stick* models depicting the information represented as bars in *M*–*O*. *P*, entry site (a), tunnel site (b), exit site (c), figure key (d). For more detail on figure *P* refer Figure 5*C* (6).

To gain insight into this possibility, we analyzed the statistical distribution of B-factors in different apo structures at pH 5.0, 7.0, and 9.0, which were solved at comparable resolutions of 2.63, 2.20, and 2.45 Å, respectively. We plotted the B-factor of the backbone [--C_*α*_-(C=O)-N--] as well as the whole residue (backbone and side chain). The average whole-residue B-factors of pH 5.0, pH 7.0, and pH 9.0 structures are 13.87, 26.42, and 14.73 (Å)^2^, respectively. Similarly, the average backbone atom B-factors for pH 5.0, 7.0, and 9.0 were found to be 13.39, 25.56, and 14.17 (Å)^2^, respectively. The B-factor plot and B-factor spectrum image for these data visually depict the difference in protein flexibility with changing pH. The protein is least flexible at acidic pH, most flexible at neutral pH, and has intermediate flexibility at basic pH (Fig. 6, *A*–*F*). We also devised a more specific B-factor plot to analyze flexibility of the catalytic tunnel substrate binding and lining residues. As per our nomenclature mentioned previously for describing the apo Smlt1473 crystal structure, we plotted the B-factor of entry, tunnel, and exit site residues (Fig. 6, *G*–*I*). The B-factor distribution was also depicted in the stick model colored by B-factor spectrum (Fig. 6, *J*–*L*). The entry site residues have an average B-factor of 15.34, 26.06, and 14.32 (Å)^2^ for pH 5.0, 7.0, and 9.0, respectively; tunnel binding residues have an average B-factor of 11.70, 25.23, and 9.79 (Å)^2^ for pH 5.0, 7.0, and 9.0, respectively; and exit site residues have average B-factor of 12.49, 27.84, and 9.02 (Å)^2^, respectively. Thus, one mechanism of varying substrate accessibility as a function of pH is in the catalytic tunnel flexibility, which correlates with specific activity; in other words, the greater the flexibility in the catalytic tunnel region (in terms of B-factors), the greater the spread of specific activity range observed around a pH optimum and therefore greater accessibility of the substrate to the catalytic tunnel region. Consistent with this idea, the above B-factor trends correlate with previously reported specific activity measurements for Smlt1473 as a function of substrate and pH. In particular, Smlt1473 has maximum activity for polyGlcA at pH 7.0 as well as the greatest spread in activity with regards to pH (>75% of maximum specific activity retained between pH 5.5 and pH 8.0) and also the largest observed B-factor. However, at pH 5.0 and 9.0, the experimental spread in specific activity is considerably narrower relative to pH 7.0, with correspondingly lower B-factors observed (5, 6). To remove any bias that would arise from crystal packing, we also performed B-factor scaling by dividing individual B-factors of the molecule with average B-factor. Our comparative analysis of scaled B-factor values of entry, tunnel, and exit site residues at pH 5.0, 7.0, and 9.0 has furthered our observation and revealed some interesting facts that have refined our interpretation (Fig. 6, *M*–*O*). Of all 24 substrate-interacting residues, pH 5.0, 7.0, and 9.0 structures have, respectively, 9 (37.50%), 12 (50.00%), and 3 (12.50%) residues with the highest B-factor values (Fig. 6, *M*–*P*).

At the entry site, pH 5.0, 7.0, and 9.0 have three (Q52, K56, D63), one (R312), and two (R218, H221) residues, respectively (Fig. 6, *M* and *P* (a)). The pH 5.0 and 9.0 apo structures possess higher flexibility at the entry site than at pH 7.0. Of interest, the pH 5.0 structure has fluctuations on the left side of the entry site, which is opposite to the poly-HA (pH 5.0 specific) path of egress (Figs. 5 and 6*P* (a)). In contrast, pH 9.0 has these fluctuations on the right side of the entry site, which is opposite to the poly-ManA (pH 9.0 specific) path of egress (Figs. 5 and 6*P* (a)). For poly-ManA activity, the mutations R218L and H221F lead, respectively, to minimally reduced and enhanced specific activity (Fig. 5*C* (c)). For pH 5.0–specific poly-HA, all of these mutations lead to significant reduction in specific activity (Fig. 5*C* (d)). For pH 7.0–specific polyGlcA, except for R312 the rest of residues show significant loss of specific activity (6). The effect of Q52, K56, and D63 mutations have not been tested for any substrate (Fig. 6*P* (a)).

At the tunnel site, pH 5.0 and 7.0 have three (N55, W119, Y222) and four (Y38, Y39, W171, Y225) residues, respectively, with the highest atomic fluctuations. Here the residues are not aligned to a specific side of the tunnel but are clustered as a diagonal pair contributing to both the right and left sides of the catalytic tunnel (Fig. 6*P* (b)). For pH 7.0–specific polyGlcA, the mutations W71A and Y225F lead to enhanced catalytic activity, suggesting no role in substrate stabilization. For poly-HA, these mutations are most detrimental as these residues lie directly in its path of egress, whereas for polyManA, these mutations had effects similar to polyGlcA, resulting in specific activity enhancement (Fig. 5*C* (c)). The effect of Y38, Y39, N55, and W119 mutations have not been tested.

At the exit site, pH 5.0, 7.0, and 9.0 have three (R163, N166, E212), seven (K42, N110, Q112, Y115, K162, N167, H168), and one (R215) residue, respectively, with the highest atomic fluctuations. The distribution of the highest fluctuating residues is again localized according to pH. The pH 5.0 has the highest fluctuating residues at the [+2, +1] subsite (N166, R215) and ahead (R163). For pH 7.0, such residues are at the [+2, +1] subsite (R163, N167, H168) and on the left side (K42, N110, Q112, Y115) (Fig. 6*P* (c)). For pH 9.0, the only such residue R215 lies at the +1 subsite and contributes to the right side as its entry site counterparts R218 and H221 (Fig. 6*P* (c)). For poly-HA, mutation of K42, Y115, K162, and R163L result in moderate to intermediate loss of activity as compared with previous mutations (W171A, R218, H221). For polyGlcA, except for enhancing mutation Y115A, all of these mutations resulted in significant loss of activity, whereas for polyManA, except for the moderately enhancing mutation K42L, other mutations resulted in moderate to significant loss in specific activity. The effect of N110, Q112, N166, and E212 (58% of wild-type activity against alginate (8)) mutations have not been tested for all substrates.

We observed opposite effects of certain mutations on polyGlcA and polyManA specific activity at the entry (H221F, R312L) and exit (K42L, Y115F) sites but not at the tunnel site (Fig. 6*P*). However, the only difference between these two substrates is at the C3 position, which have orientations that are epimers of each other. This will lead polyGlcA to not have polyManA/HA-specific interaction (R312) and gain of ManA specific and polyHA-specific interaction (H221). Thus, the mode of polyGlcA binding will be different at the entry and exit sites and will be in between that of polyManA and polyHA. Alternatively, we compared only the residues that form noncovalent interactions with the substrates. We found that pH 5.0, 7.0, and 9.0 have, respectively, two (N55, Y222), four (Y38, Q112, N167, H168), and three (R215, R218, H221) residues with the highest atomic fluctuations at the [−1, +1] subsite. This generalization implicates the pH 7.0 apo structure catalytic tunnel to be the most flexible and rationalizes the less numerous and weaker interactions for the ManA cocrystallized pH 7.0 structure compared with pH 5.0, as discussed in the previous section.

### B-factor analysis of ManA-complexed Smlt1473 structures at pH 5.0 and 7.0

**Figure 7:**
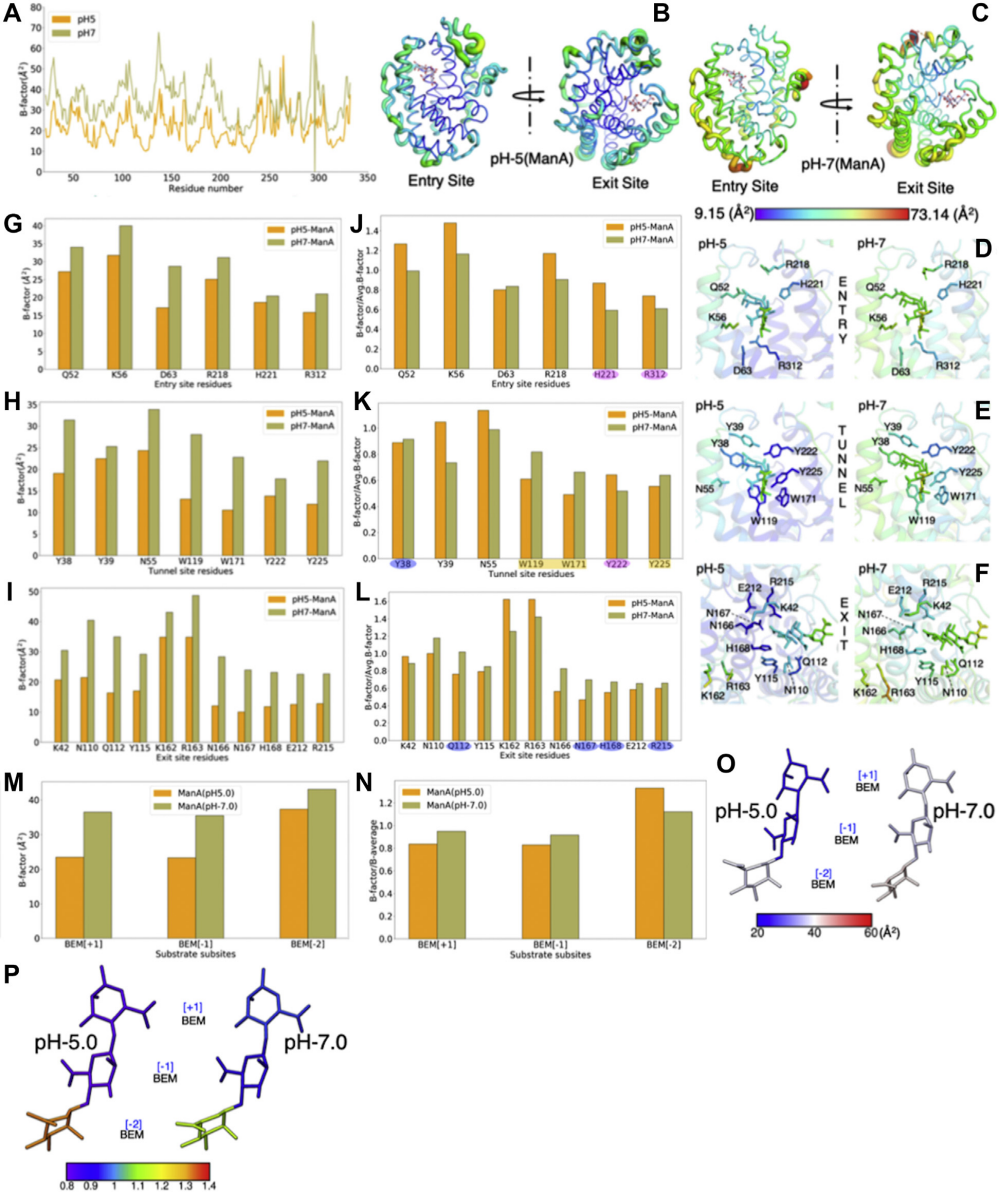
**B-factor distribution of Smlt1473(WT) ManA-complexed structures at pH 5.0 and 7.0.** Residue-wise B-factor superposition of all amino acid residues of ManA-complexed WT structure at pH 7.0 and 5.0 (*A*). B-factor putty cartoon model showing the entry and exit sites of ManA-complexed structure at pH 5.0 (*B*), pH 7.0 (*C*). B-factor spectrum bar showing distribution of color code used to color the putty models; *violet* and *red* depict the minimum and maximum values of B-factor, respectively. WT-ManA-complexed structure at pH 5.0 and 7.0 showing the substrate-interacting residues in catalytic tunnel at entry site (*D*), tunnel site (*E*), and exit site (*F*). The substrate and vicinity residues are shown as *stick* model and colored according to B-factor. The corresponding B-factor distribution for each site is shown as bar plot (*G*–*I*). The normalized B-factors (B-Factors/Avg. B-factors) are shown for entry (*J*), tunnel (*K*), and exit site (*L*). The residues interacting directly with substrate at +1 subsite (Y38, Q112, N167, H168, R215) are encircled with *purple shaded ovals*, and rest (H221, R312, Y222) with *magenta shaded oval*. The catalytic tunnel’s core maintaining aromatic residues (W119, W171, Y225) are marked under a *rectangular orange block*. The substrate residue’s B-factor are modeled as *stick* and colored with three color spectrums (*blue*, *white*, *red*) (*O*), the corresponding bar plot is represented (*M*). Similarly, the normalized B-factors are modeled as *colored stick* for bound substrate. The spectrum color bar (VIBGYOR, 0.1 unit/color) depicts the degree of atomic fluctuations (*P*). A corresponding bar plot represents the atomic fluctuation distribution as a function of subsites (*N*).

The Smlt1473 ManA-complexed structures at pH 5.0 and 7.0 are solved at resolution 2.31 and 2.43 Å, respectively. We have performed three levels of B-factor comparisons: (i) at the level of the whole molecule (Fig. 7, *A*–*C*), (ii) at the level of the catalytic tunnel residues (Fig. 7, *D*–*F*), and (iii) at the level of the scaled B-factor to remove the crystal contact and data resolution bias (Fig. 7, *J*–*L*). At the molecular level, pH 7.0 bound structure shows a high degree of fluctuation (Avg. B-factor = 34.42 Å^2^) compared with pH 5.0 (Avg. B-factor = 21.95 Å^2^) bound structure (Fig. 7, *A*–*C* and Table 1). This trend in B-factor fluctuations as a function of pH is consistent with apo structures. However, in contrast to apo structures (solved at comparable resolution), the average B-factor in bound structures at pH 5.0 and 7.0 has increased by nearly eight units (Table 1). The residue-wise B-factor (colored with B-factor spectrum [Fig. 7, *B*–*F*] and represented as putty [Fig. 7, *B* and *C*], cartoon, and stick [Fig. 7, *D*–*F*] models, bar plots [Fig. 7, *G*–*I*]) reveals that, at pH 7.0, the ManA binding is not as strong as at pH 5.0. As previously, it again explains why there is a resolvable electron density only up to three ManA rings in the hexa-ManA-complexed pH 7.0 structure.

Among the bound structures, the scaled B-factor of the entry site residues accounts for higher atomic fluctuations (Fig. 7*J*) at pH 5.0. This result is consistent with the analysis of apo structures at pH 5.0 and pH 7.0 (Fig. 6). The continuation of the similar B-factor trend among apo and substrate bound structures at the entry site directly implicates toward a structural mechanism of acquiring bulkier residues (HA) at low pH. At the tunnel site, the residues contributing to the highest atomic fluctuations are tilted toward pH 7.0 (Y38, W119, W171, Y225) than pH 5.0 (Y39, N55, Y222) bound structures. Apart from the highly interacting Y38, the aromatic residues (W119, W171, and Y225) maintain the catalytic tunnel core, having higher atomic fluctuations at pH 7.0 bound structure and resulting in high flexibility at the tunnel core for pH 7.0 bound structure (Fig. 7*K*). At the exit site, the residues involved in direct H-bond and Van der Waals interactions with substrate at the +1 subsite have high (N110, Q112, Y115, N166, N167, H168, E212, R215) atomic fluctuations for pH 7.0 bound structure (Fig. 7*L*), in contrast to pH 5.0 bound structure. These observations imply that substrate binding at the [−1, +1] subsite is much tighter in terms of atomic fluctuations for the pH 5.0 bound structure. The B-factor (normal and scaled) comparison of bound substrate residues at pH 5.0 and 7.0 further supports the above observations and interpretations (Fig. 7, *M*–*P*). The trend in normalized B-factor for bound substrates at the [+1, −1] subsite corresponds with the substrate-binding residues.

### B-factor comparison between apo and ManA bound pH 5.0 and 7.0 structures

**Figure 8:**
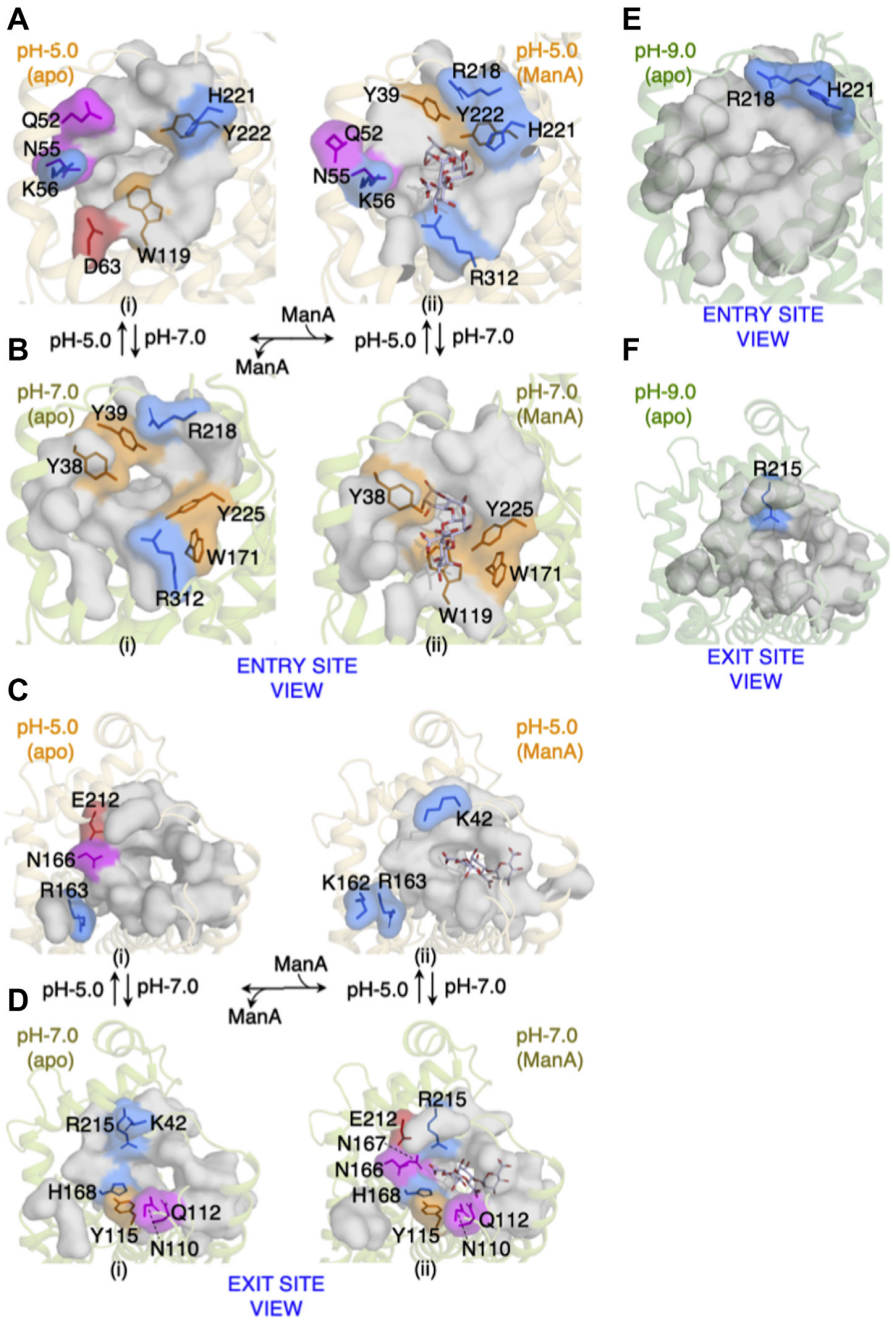
**Comparison of B-factor distribution among apo and substrate bound structures for residues lining the catalytic tunnel.** The entry and exit site residues with the highest atomic fluctuations among apo, substrate bound WT pH 5.0 and 7.0 structures are shown. The residues are represented as *colored surfaces* and *stick* model for WT pH 5.0 apo and tetra-ManA bound structure at entry site (*A*), WT pH 7.0 apo and tri-ManA bound structure at entry site (*B*), WT pH 5.0 apo and tetra-ManA bound structure at exit site (*C*), WT pH 7.0 apo and tri-ManA bound structure at exit site (*D*). *Colored surfaces* are according to residue types: *magenta*, polar uncharged; *orange*, aromatic; *blue*, basic amino acids; *gray*, low atomic fluctuations. For WT pH 9.0 apo structure, the residues with the highest fluctuations as compared with WT pH 5.0 and 7.0 apo structures (*E* and *F*). This figure is based on data from Figures 6, *M*–*O* and 7, *J*–*L*.

For comparing apo and ManA-complexed structures at pH 5.0 and 7.0, we have modeled the catalytic tunnel as a surface and the rest of structure in illustrative form. For all structures, we have represented “entry site view” and “exit site view,” which display the entry site, tunnel site, and exit site residues. For each model, the residues with maximum scaled B-factor are colored according to residue type such as blue (basic), red (acidic), magenta (polar uncharged), orange (aromatic), and gray (other residues with comparably low scaled B-factor); the colored residues are also represented as stick (Fig. 8, *A*–*F*). We found that the atomic fluctuations are clustered differently for pH 5.0 and 7.0 ManA-complexed structures (Fig. 8, *A*–*F*).

The pH 7 apo structure at the entry site has the following residues with high atomic fluctuations: Y38, Y39, W171, R218, Y225, and R312 (Fig. 8*B* (i)). Y38 and Y39 are part of the N-terminal lid loop constituting the *R3* surface of the entry site, R218 constituting the roof *R2* surface of the entry site, W171 and Y225 forming the *iRW* surface of the entry site, and R312 forming the *F1* and *F2* surface of the entry site (Fig. 2*H*). At the exit site (Fig. 8*D* (i)), the pH 7 apo structure has continuation of the high atomic fluctuations in the same left side from the exit site view (*i LW* and *ii LW* surface) and includes residues such as K42, Y115, H168, and R215. N110 and Q112 are part of the loop (L3) forming the ii*RW* surface (Fig. 2*G*). Upon substrate binding, the high atomic fluctuation at the entry site shifts toward the right side of the wall (*iRW*; W171, Y225) and floor (*F3*; W119) (Fig. 8*B* (ii)). Similarly, at the exit site the high atomic fluctuation forms a crescent contributed by Q112, N110, Y115, H168, N166, N167, E212, and R215 (Fig. 8*D* (ii)). This results in a unidirectional and continuous patch of flexible residues from the entry to exit site for ManA bound structure at pH 7.0 (Fig. 8, *B* (ii) and *D* (ii)).

For the pH 5.0 apo structure, the residues with comparably high atomic fluctuations at the entry site include Q52, N55, K56 (*iLW* surface), D63 (*F1* surface), W119 (*F3* surface), Y222, and H221 (*R3* surface) (Figs. 2*H* and 8*A* (i)). At the exit site, the residues N166 and E212 forming the *iiLW* surface show the highest atomic fluctuation (Figs. 2*G* and 8*C* (i)). Upon substrate binding, the entry site view of the pH 5.0 ManA-complexed structure presents a contrasting patch of residues in comparison with the entry site view of the pH 7.0 structure (Fig. 8, *A* (ii) and *B* (ii)). At the exit site, the fluctuation of K42 (constituent of the N-terminal lid loop and forms the ii*RW* surface), K162, R163 (*iiLW* surface) increased as compared with the apo structure (Fig. 8*C* (ii)). The atomic fluctuation of the +1 subsite substrate-interacting residues N110, Q112, N166, N167, and H168 decreased comparably.

Collectively, the comparison of the normalized B-factor of bound structure with apo structure at a particular pH confirms that the clustering of pH-dependent atomic fluctuations is largely intrinsic to the enzyme and binding of substrate just refines them further (Fig. 8). Together these observations support the idea that atomic fluctuations in the enzyme catalytic tunnel are preconfigured and pH helps to tune the optimal fluctuation resulting in pH-dependent optimal binding and cleavage.

### Electrostatic surface potential maps as a function of pH correlates with pH-specific substrate binding and specificity

**Figure 9:**
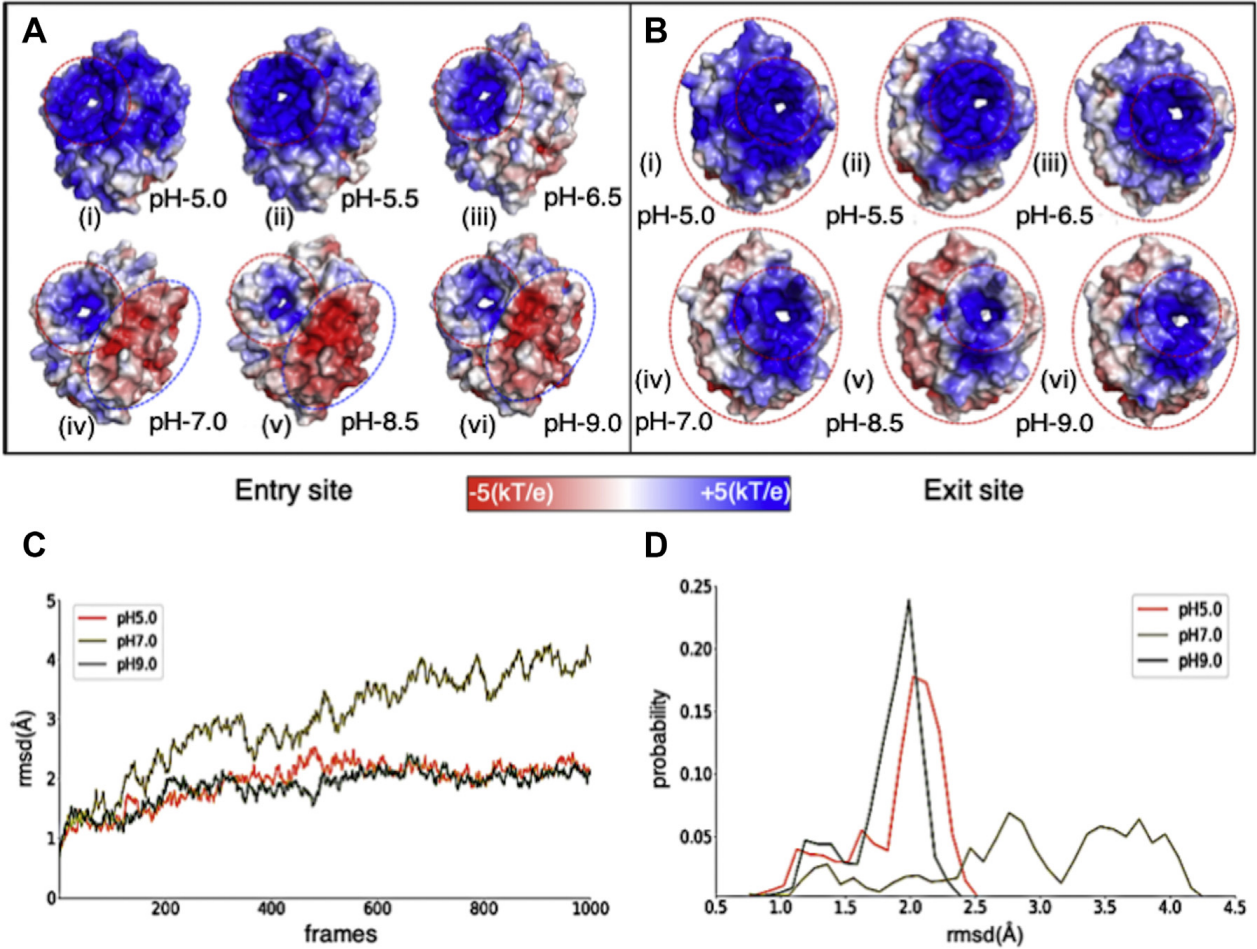
**Comparison of electrostatic surface model of Smlt1473 WT apo structures (solved at different pH).***A* (i-vi), entry site and *B* (i-vi) exit site, calculated at different pH. The titration states at different pH were assigned by invoking command line version of pdb2pqr. The electrostatic models are generated by APBS plugin of PyMol by using pdb2pqr outputs, .pqr file, and APBS input template. The color scale of electrostatic potential ranges from −5 kT/e (*red*/electronegative) to +5 kT/e (*blue*/electropositive). CpHMD calculations of tetra-ManA-complexed Smlt1473 (WT, pH 5.0) at pH 5.0, 7.0, and 9.0 (*C* and *D*).

In the above discussion, we have very briefly stated the effect of pH-driven electrostatic interactions in substrate binding. In this section we will detail our results of electrostatic modeling of apo structure as a function of pH. This modeling helped us to ascertain the electrostatic effects of pH variation on substrate binding and catalysis. The detail of the procedure can be found in the Experimental procedures section. We have displayed the entry site (Fig. 9*A* (i–vi)) and exit site (Fig. 9*B* (i–vi)) at pH 5.0, 5.5, 6.5, 7.0, 8.5, and 9.0. We observe significant differences in net charge as a function of pH; at the entry site (Fig. 9*A*), positive charge decreases gradually as the pH increases. As the pH becomes basic, the region of positive charge is confined toward the tunnel (Fig. 9*A* (iv–vi)) and an interesting emergence of a negatively charged surface occurs at the right-hand side of the tunnel (Fig. 9*A* (iv–vi)). In Figure 9*B* (iv–vi), the smaller circle represents the exit site and the space between the bigger oval and the smaller circle shows a gradual decrease in electropositivity and follows similar trends as the entry site. These observations provide a key insight regarding the question of how pH-dependent substrate specificity occurs for Smlt1473. PolyHA is a (1→4)-linked polymer of a dimer [β-D-Glucuronic acid (1→3) N-acetyl-β-D-glucosamine], where only β-D-glucuronic acid possesses a carboxylic group, giving poly-HA a lower negative charge density in comparison with other substrates like polyGlcA and polyManA, which contain only uronic acids and thus have higher negative charge density. Thus, in comparison with other polyuronide substrates, poly-HA requires an extended electropositive surface (Fig. 9*A* (i and ii)) for binding; the prior enzyme kinetic data also support this idea as mutation of any residue in the tunnel decreases or eliminates poly-HA turnover (Fig. 5*C* (b and d)) (6). For poly-ManA, whose optimal pH for activity is 9.0, we have been able to trap tetra-ManA and hexa-ManA (three units) in a bound state to the wild-type enzyme at pH 5.0 and pH 7.0, respectively. The electrostatic models have already shown an extended electropositive surface at pH 5 (Fig. 9*A* (i)); thus, bound substrate at pH values where no turnover is observed raises the interesting possibilities of whether (i) at nonoptimal pH the specific electrostatic binding requirement for substrate turnover is not realized, or (ii) at nonoptimal pH strong substrate binding affinity negatively affects catalytic turnover. In other words, the pH dependence of specific activity is necessary to provide a suitable electrostatic microenvironment for substrate catalysis in which both binding strength and substrate configuration are important. This interplay of substrate and enzyme electrostatics as a function of pH is the result of molecular adaptation of Smlt1473 to translocate the substrates through the catalytic tunnel and position the substrate for effective turnover.

### Constant pH molecular dynamics calculations combine the dynamic view of B-factor and static view of electrostatics in substrate acquisition and enzyme catalysis

The large-scale atomic fluctuation confirmed by both B-factor analysis (Figs. 6 and 7) and constant pH molecular dynamics (CpHMD) simulation (Fig. 9, *C* and *D*) suggest that, at pH 7.0, the atomic fluctuations are high. Our CpHMD result suggests that, for mannuronate, 9.0 is the most optimal pH for catalysis, as like pH 7.0 it does not explore conformational states significantly different from the crystal structure (Fig. 9*D*). Of interest, the pH 5.0 structure has shown atomic fluctuations similar to pH 9.0 (Fig. 9*C*), which we have already confirmed during B-factor analysis (Figs. 6 and 7). However, the high binding affinity for ManA at pH 5.0 confirmed by substrate pH trapping, substrate normalized B-factor analysis (Fig. 7, *J* and *K*) precludes catalysis at pH 5.0. Thus, at pH 9.0, the substrate binding energy and atomic fluctuation are most optimal for ManA catalysis.

### Smlt1473 is structurally unique among PL-5 family to acquire substrate differing in stereochemical composition

**Figure 10:**
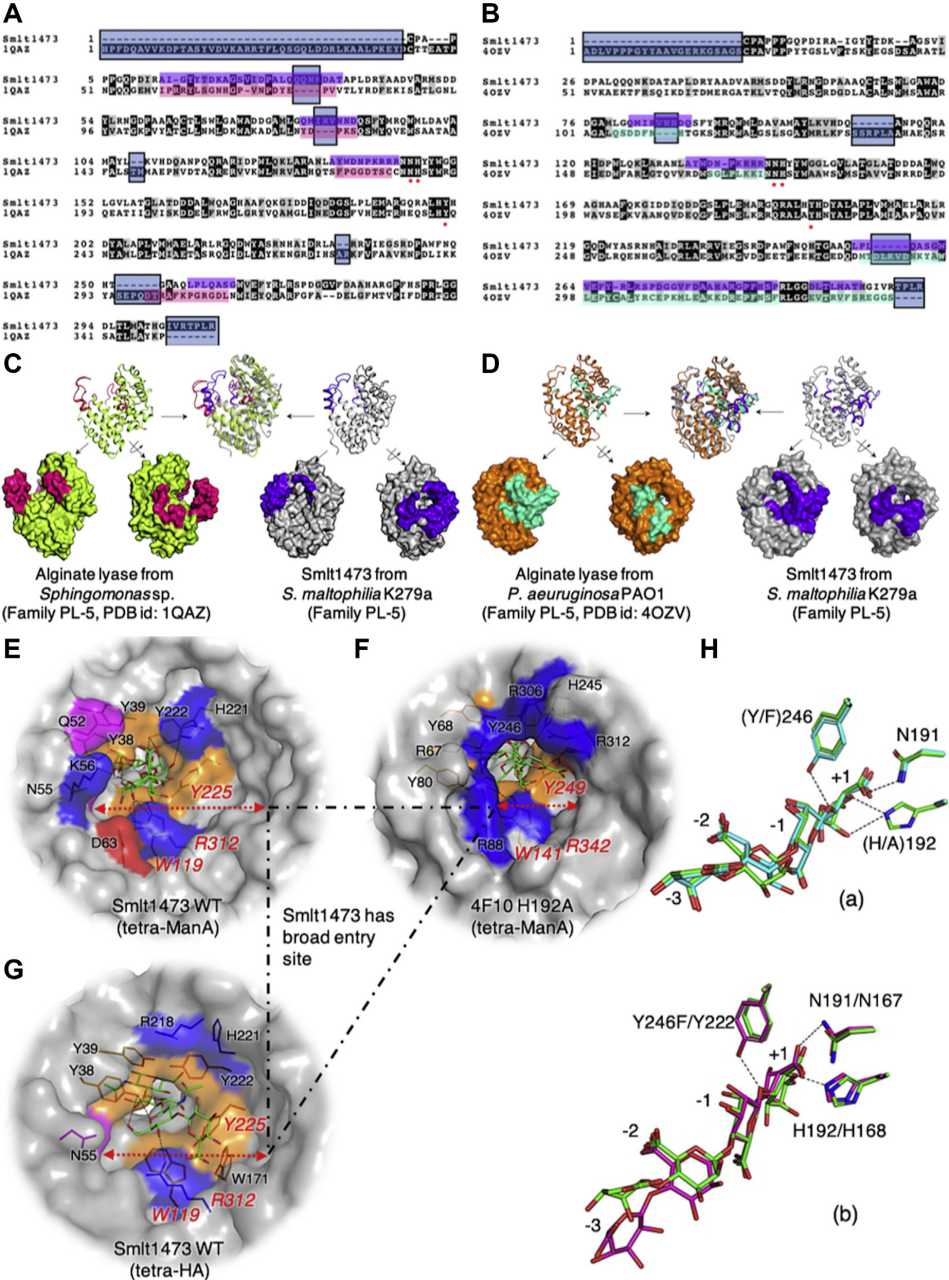
**Sequence and structural analysis of Smlt1473 and other PL-5 proteins.** Sequence alignment between Smlt1473 and 1QAZ (*Sphingomonas* sp.) (*A*), Smlt1473 and 4OZV (*P. aeruginosa* PAO1) (*B*), structural alignment and comparison of substrate entry and exit surface for *A* (*C*), for *B* (*D*). Enzyme–substrate interaction at entry site of Smlt1473 wt complexed with ManA at pH 5.0 (fourth unit is modeled) (*E*), PL-5 polyManA lyase active site mutant (H192A) complexed with tetra-ManA (*Sphingomonas sp.* A1) (*F*), Smlt1473 wt pH 5.0 docked with tetra-HA (*G*). Superposition of complexed substrates of H192A (PDB ID: 4F10) and Y246F (PDB ID: 4F13) mutants of PL-5 from *Sphingomonas sp.* A1, [*H* (a)] and *E* and *F* [*H* (b)], Smlt1473 bound substrate’s fourth BEM unit is modeled.

Among the PL-5 family to which Smlt1473 belongs, there are only two PLs characterized structurally, one of which has substrate cocrystallized (13). One of the *apo* structures (Protein Data Bank (PDB) ID: 4OZV) has an active site region in a tunnel similar to Smlt1473, whereas in the other the tunnel forms only after substrate binding (13). Smlt1473 falls in the group where tunnel-like active site architecture is present in the *apo* as well as substrate-bound states. To draw differences between the available PL-5 structures and Smlt1473 structures, we performed sequence as well as structural alignments. We find distinct differences in sequence and structure that distinguish Smlt1473 from other PL-5 members. One major difference is at the substrate entry site, where the reduced size of the inserted loop in the other two PLs narrows access to the entry site; in Smlt1473, a significantly larger entry site loop is observed. This provides one key structural difference as to why Smlt1473 can accommodate structurally and chemically different substrates. The larger loop allows greater flexibility to accommodate diverse substrates, where other PLs cannot owing to spatial limitations of the entry site loop (Fig. 10, *A*–*D*). The spatial limitation of other PL-5 family members *versus* Smlt1473 is also evident when comparing the substrate entry tunnel among substrate bound PL-5 structures (Fig. 10, *E* and *F*). The incoming substrate in case of *Sphingomonas* sp. interacts directly with both sides of the active site wall (Fig. 10*F*), whereas in case of Smlt1473 there is significant additional unoccupied, noninteracting space to the right of the active site tunnel, which is utilized for binding poly-HA (Fig. 10*G*). The structural superposition of the substrates from *Sphingomonas* sp. H192A (green stick) and Y246F (cyan stick) mutant structure shows no significant structural differences (Fig. 10*H* (a)). But, when the Y246F mutant bound substrate is superimposed with wild-type Smlt1473 (pH 5.0) complexed tera-ManA structure, a clear difference is observed at the [−2, −3] subsite. In Smlt1473, the mode of substrate binding is not only distinct but more relaxed in terms of specific substrate–tunnel contacts (Fig. 10, *F* and *H* (b)). Thus, these results provide further evidence of the unique evolution of structural flexibility in the tunnel and divergence in loop structure for Smlt1473 *versus* other PL-5 family members and the unique structural features that enable Smlt1473 to accept multiple, divergent polysaccharide substrates.

## Discussion

Our work reveals several interesting new mechanisms that distinguish Smlt1473 from other PLs. In contrast to other PL-5 family members, Smlt1473 catalytic architecture is not an open cleft but rather a tunnel formed by interactions involving a significantly longer N-terminal lid loop. Furthermore, a wider tunnel entry site favors the stereochemical accommodation of multiple substrates *versus* the constrained active site architecture in other PL-5 family members (Fig. 10). The B-factor analysis presented provides direct correlation between protein flexibility within the catalytic tunnel and experimentally determined enzyme kinetic parameters measured for a given substrate–pH paring (Figure 6, Figure 7, Figure 8).

Our electrostatic models (Fig. 9, *A* and *B*) suggest that Smlt1473 possesses a pH-tuned, complementarily charged surface for binding and catalyzing turnover of substrates with varying negative charge densities. Our ability to trap ManA (optimum catalytic turnover pH 9.0) at pH values 5.0 and 7.0 in wild-type complexed structures provides further direct evidence to support this mechanism (Fig. 3). To our knowledge, this is the first report of “pH trapping” to generate wild-type enzyme–substrate complexes among CAZymes. The comparison of substrates in enzyme–substrate complex structures (Smlt1473–4ManA and Smlt1473–4HA) provides a mechanistic basis for defining the mode of substrate binding, the path of egress, and understanding why prior experimental mutations described in the catalytic tunnel enhance or diminish substrate turnover. For both substrates, sugar ring puckering is conserved only at the [−1, +1] subsite, whereas it differs at other subsites restricting the orientation of ManA and HA to enter from the left and right sides of the catalytic tunnel, respectively. This mode of substrate entry correlates with prior experimental data for residues mutated in the catalytic tunnel. For ManA, mutations in the right and left sides of the tunnel diminish and enhance the specific activity, respectively. For HA, mutations on the right side of the tunnel are most detrimental to substrate turnover (Fig. 5).

Overall, our work collectively provides a detailed structural model for the unique, pH-driven substrate specificity of Smlt1473. The structural information obtained from this work can form the basis for potential inhibitor design relevant to biofilm formation and provide templates for future protein engineering approaches to enable various biotechnological and health care applications. In addition, gram-positive pathogens such as *Streptococcus* produce hyaluronidases, and the role they play in bacterial virulence is an area of active research (21). For gram-negative bacteria such as *S. maltophilia*, hyaluronidase activity has not been described, making Smlt1473 unique among bacterial hyaluronidases in terms of function. Future microbiological studies will be necessary to determine what roles Smlt1473 plays in processes such as biofilm formation and possible virulence. Nonetheless, our results point to an interesting instance of potential convergent evolution in which multiple substrates and attributable functions have evolved within a single enzyme.

## Experimental procedures

### Overexpression and purification of Smlt1473 wild-type and mutants

Smlt1473 wild-type and mutants were cloned in pET28a(+) as BamHI-XhoI inserts, transformed into *Escherichia coli* BL21(LEMO) cells, and plated on selective kanamycin (50 μg/ml) and chloramphenicol (50 μg/ml) LB plates. Individual colonies were isolated in 40 ml of selective LB broth and incubated overnight at 37 °C, 220 rpm in Innova 42 shaker-incubator (New Brunswick) to generate saturated primary cultures. A volume of 10 ml of saturated culture was used to seed 1 l selective LB cultures for large-scale production. One-liter cultures were incubated at 37 °C, 220 rpm in Innova 44 shaker-incubator (New Brunswick) and monitored until an *A*_600_ of 0.4 was reached. At *A*_600_ = 0.4, the cells were quickly transferred to a shaking incubator set at 18 °C, incubated at 18 °C and 220 rpm until an *A*_600_ = 0.6 was reached. The culture was induced by adding 1 ml of 1 M IPTG to achieve a working concentration of 1 mM IPTG in each large-scale culture, and bacterial growth was maintained for 16 h at 18 °C and 220 rpm. After 16 h, the cultures were harvested by centrifugation at 8000*g* for 15 min, decanting the supernatant, and storing bacterial cell pellets at −80 °C. For SDS-PAGE analysis, 1-ml culture samples were lysed by boiling in 50 μl of gel loading dye (for 1 ml, 950 μl Laemmli sample buffer and 50 μl of β-mercaptoethanol) at 95 °C for 5 min, then run on a SDS-PAGE (15%) gel with mid-range protein marker (14–95 kDa). For purification, pellets stored at −80 °C were taken and resuspended in lysis buffer (IMAC buffer A) containing 100 mM Tris-HCl (pH 7.5), 500 mM NaCl, 10% v/v glycerol, 10 mM imidazole, and 1 mM β-mercaptoethanol. Resuspended pellets were sonicated at 40 W on ice using a tip sonicator with 10 s ON and 15 s OFF cycle times and total processing time of 15 min. The sonicated cell lysate was clarified by centrifuging at 17,000*g* for 1 h at 4 °C. The supernatant was collected and the pellet was discarded, and an additional wash of 10% w/v of streptomycin sulfate was introduced to precipitate nucleic acids and other residual cellular debris, then centrifuged at 30,000*g* for 30 min at 4 °C. Protein present in cell lysates was purified using immobilized metal ion affinity chromatography (IMAC) on a GE AKTA pure 25M FPLC system. The cell lysate was loaded at 1.0 ml/min over a 5-ml GE HIS-TRAP fast flow Ni-Sepharose column pre-equilibrated with IMAC buffer A. After sample application, four column volumes of 40 mM imidazole buffer was used for an initial wash followed by two column volumes intermediate wash of 80 mM imidazole buffer. The protein was eluted with 8% to 100% gradient of IMAC buffer B, which contains 100 mM Tris-HCl (pH 7.5), 500 mM NaCl, 10% v/v glycerol, 1 M imidazole, and 1 mM β-mercaptoethanol. The purity of the eluted fraction was checked by running an SDS-PAGE (15%) gel electrophoresis. Pure fractions were pooled and dialyzed against 2 l of dialysis buffer (20 mM Tris-HCl, pH 7.5, 100 mM NaCl, and 10% v/v glycerol) with three total exchanges where each exchange lasted for 3 h. Dialyzed samples were concentrated to 6 to 8 mg/ml and desalted in three different pH buffers: pH 5 (20 mM sodium citrate, 50 mM NaCl), pH 7.0 (20 mM sodium cacodylate, 50 mM NaCl), and pH 9 (20 mM Tris-HCl, 50 mM NaCl) using Bio-Rad Econo-Pac 10 DG Desalting Columns. Samples were aliquoted, flash frozen, and stored at −80 °C for long-term use.

### Crystallization

The hanging drop vapor diffusion method was used to set initial crystallization trials using commercial screening kits (Hampton Research, Anatrace). Initial conditions favorable for crystal growth from these screens included 0.2 M lithium sulfate monohydrate, 0.1 M Bis-Tris, pH 5.5, 25% w/v PEG-3350; 0.2 M ammonium sulfate, 0.1 M Bis-Tris, pH 5.5, 25% w/v PEG-3350; 0.2 M ammonium sulfate, 0.1 M sodium cacodylate trihydrate, pH 6.5, 30% w/v PEG-8000; 0.2 M ammonium sulfate, 0.1 M sodium cacodylate-HCl, pH 6.5, 30% w/v PEG-8000; 0.2 M lithium sulfate monohydrate, 0.1 M Tris-Cl, pH 8.5, 30% w/v PEG-4000; 0.2 M ammonium sulfate, 0.1 M Tris-Cl, pH 8.5, 25% w/v PEG-3350. To improve crystal quality and to obtain crystals at different pH values, we prepared a focused crystallization matrix by optimizing salt, buffer, and precipitant concentrations around the initial conditions. From this focused screen, we obtained diffraction quality crystal at over a broad spectrum of pH values (4.0, 5.0, 5.5, 6.5, 7.0, 8.5, and 9.0) for both wild-type and mutant Smlt1473.

### X-ray diffraction data collection, data processing, structure solution and refinement

Protein crystals were briefly soaked in 1 μl of cryopreservative (ethylene glycol diluted to 20% v/v with mother liquor) solution, then picked and centered into the X-ray beam path of BRUKER-PROTEUM X-ray diffractometer (home source at NISER). A continuous stream of liquid nitrogen at 100 K was used to avoid radiation-induced damage. Crystals were checked for diffraction quality; well-diffracting crystals were used for determining unit cell parameters. The data collection strategy was designed according to the unit cell parameters; data collection was performed by combining geometrically different runs (φ-scan, ω-scan) to obtain statistically acceptable datasets. The raw X-ray reflection images obtained from data collection was processed using PROTEUM 2 software from BRUKER. First, the sets of X-ray images from different runs were harvested and the reflections were indexed to determine the bravais lattice, followed by the cell parameters refinement. Second, the intensity of the reflections was determined by integration, and finally the reflections were scaled. After scaling, the data quality was checked using the XPREP program suit of PROTEUM 2, the space group was assigned, and scaled output was written to the file. Several programs in CCP4 (22) suite were used during the course of structure determination. The MATTHEWS(CCP4) was used to determine the Matthew’s coefficient and number of molecules in asymmetric unit (23, 24). The merged and scaled data were converted to *mtz* format by using SCALEPACK2MTZ (CCP4), and 5% of reflections were kept aside for cross-validation purposes during refinement. Next, the structure was determined by performing molecular replacement using PHASER(CCP4) (25); for Smlt1473 wild-type crystal data at pH 8.5, the structure of alginate lyase A1-III (PDB ID: 1QAZ) was used as the search model. For other structures, the Smlt1473 wild-type structure at pH 8.5 served as the search model. After molecular replacement, rigid body refinement was first run using REFMAC5(CCP4) (26), followed by a restrained refinement, and electron density was manually inspected and the model was build using COOT (27). Structure was determined by iterating model building and restrained refinement until refinement parameters R_work_ and R_free_ reached below or equal to agreeable limits. Table 1 and Table S1 show the data collection and refinement statistics.

### Electrostatic modeling of Smlt1473 apo structure as function of pH

For electrostatic calculations, first the *pqr* models containing atomic charge and radii and corresponding APBS input templates were generated by using pdb2pqr (28, 29, 30). For input *pdb* files, pdb2pqr calculations were done with PARSE as force field, PROPKA for pKa calculations at respective pH values (5.0–9.0), and for generating H-bond, contacts, and salt bridges (27). The resultant pdb2pqr outputs were used as input for electrostatic calculations performed by PyMol APBS plugin with the following specifications: grid spacing, 0.50; electrostatic potential range, +5.0 to −5.0 Ke/T. The solvent excluded electrostatic potential surface maps were rendered in PYMOL for visualization and analysis.

### Docking of tetra-HA with Smlt1473 crystal structure

Ligand docking was performed using the Rosetta flexible protocol that implements low-resolution docking followed by high-resolution docking (31, 32). In low-resolution docking, the ligand, treated as rigid body, was translated around a predetermined binding hotspot followed by coupled rotation and translation. In high-resolution docking, the side chains were repacked and scoring was done with *ligand_soft_rep* energy terms and the complex was minimized employing *hard_rep* energy terms. The above protocol results in the ligand being sampled with different conformations and orientations when applied for different conformational states of the ligand. The side chains around the ligands were repacked and relaxed along with the backbone, giving rise to a sampling of protein conformational flexibility. A total of 5000 complex models were generated. To identify the most likely ligand-docked configurations, the models were sorted first based on total energy score, and then the top 20% of the sorted models were selected to screen for the top interfacial binding energy (Fig. S3, *B* and *C*). The most likely ligand configurations correspond to the model having the lowest interface energy with reasonable interactions for ligand with active site residues (Fig. 3*C* (ii)). In order to check for any conformational bias arising from using ligand from pdb file 1LXK, we also performed the above calculations with a protein-free crystal structure of hyaluronic acid (16). We superposed the HA ligands from both calculation results and did not find any significant differences aside from a slight change in sugar ring orientation (Fig. S3*A*).

### Molecular dynamics simulation of tetra-HA-docked Smlt1473 structure

To further validate the substrate pose obtained after docking, we utilized GROMACS 2020 version (33) MD simulation in explicit solvent (spc216.gro, tip3p.itp) with standard salt concentration (0.1 M) to sample the degree of conformational changes at 100-ns time scale by employing charmm36-mar2019 force field. We first started with system preparation; protein coordinate was taken from docked Smlt1473-4HA. The apo protein (Smlt1473) was then protonated at pH 5.0 *via* HTMD (34). The protein coordinates were processed with Gromac’s *pdb2gmx* module to generate protein coordinates (gro format), topology (topol.top), and restraint parameter (posre.itp) files. The ligand (4HA) was parameterized by Parachem’s CHARMM General Force Field (CGenFF) legacy V.1 (https://cgenff.umaryland.edu/). We used the above parametrization to generate the protein–ligand complex and topology. The dodecahedron simulation box’s unit cell with 12-Å periodic boundary condition was defined and further solvated. The solvated system was neutralized with a combination of counter ions and salt concentration (0.1 M). The treatment of long-range electrostatic interactions was done with PME, Verlet as cutoff-scheme (1.0 Å), and grid as a method to determine neighbor list, periodic boundary conditions were set in all directions (*i.e.*, pbc=xyz). After adding ions, the system was energy minimized without restraints with steepest descent algorithm at double precision. The minimization was concluded when maximum force converged to 0.1 kJ/mol. The minimization MD parameters (mdp) were same as adding ions expect for cutoff distance, which was now 1.2 Å. Energy minimization was followed by NVT (normal volume temperature) and NPT (normal pressure temperature) equilibrations. For proper temperature coupling (tc) during NVT equilibration (100 ps), we grouped the protein and ligand as one, and the second group included water and ions (tc-grps = Protein_4HA Water_and_ions). The extra mdp parameters included holonomic constraint on h-bonds using LINCS algorithm, V-rescale as tc algorithm, and velocities were assigned from Maxwell distribution at a temperature of 300 K. In the NPT equilibration (100 ps) Berendsen was used as the pressure coupling algorithm with no velocity generation step. During equilibrations the position restraints were on for both protein and ligand. After equilibration, 100 ns of production MD was performed. The mdp parameters were same as for NPT expect for no position restraints and adoption of Parrinello–Rahman as the pressure coupling algorithm. The MD analysis was performed using Gromacs trjconv, distance, rms, rmsf, and energy modules. Matplotlib was used to plot various MD analysis, and PyMol was used to render trajectory snapshots.

### Constant pH molecular dynamics simulation

This method of pKa prediction decouples the dynamic dependence of pK_a_ and protonation state on titratable amino acid conformation. It utilizes pH as an extrinsic thermodynamic factor, thus allowing it to be used as an input. CpHMD employs Monte Carlo (MC) sampling of protonation state, and between MC steps the system evolves like generalized Born solvated MD. However, there is no solvent equilibrium step, as MD is carried out in implicit solvent. During MC steps, for titratable groups the protonation states are chosen randomly. Transition free energy of protonation or deprotonation is calculated by following equation:

$$\Delta G=k_{B}T\left(\text{pH}-{\text{pK}}_{\text{a.ref}}\right)\ln\;10+\Delta G_{\text{elec}}-\Delta G_{\text{elec.ref}}$$

where, *k*_*B*_ is the Boltzmann constant, *T* is the temperature, pH is the calculation pH, pK_a.ref_ is the pK_a_ of reference compound, Δ*G*_elec_ is the electrostatic component of transition free energy for titratable group, and Δ*G*_elec.ref_ is the electrostatic component of transition free energy for the reference compound (dipeptide amino acids). The transition free energy has two components: electrostatic and nonelectrostatic. The electrostatic component can be calculated by the GB method, and the nonelectrostatic component can be estimated by using a reference compound of known pK_a_. The Δ*G* is used to apply Metropolis criterion to know whether the transition will be accepted or not; if accepted, CpHMD will continue with the new protonation state; otherwise, there will be no change in the protonation state (35).

In our work, we have used CpHMD in Amber MD environment (36). First, the input PDB (Smlt1473_pH-5.0_tetra-ManA) was edited and the titratable residues were renamed as ASP→AS4, GLU→GL4, HIS→HIP, and CYS (involved in disulfide bonds)→CYX. The column name HETATM specifying ligand atoms was renamed as ATOM. The ligand portion of PDB was saved as a separate pdb file. Hydrogen was added to the ligand pdb file and saved in mol2 format. We used *ANTECHAMBER* tool of Amber to parametrize the ligand by defining atom type and charge (AM1-BCC) (37). It uses GAFF force field, which is compatible with other Amber forcefields. The output ligand in mol2 format was converted back to pdb format and added to the protein pdb to get ligand-complexed pdb. The same mol2 file was also used to generate tetra-ManA ligand library *via tleap*. Topology file (parm7) and input coordinate (rst7) were prepared from ligand-complexed pdb by Amber’s *tleap* module. Sourcing leaprc.constph in *tleap* sets the required ff10 forcefield and PBradii mbondi2. For specifying ligand atom (tetra-ManA) parameters, we sourced GAFF forcefield and loaded the ligand library generated earlier. After saving the file generated by *tleap*, we generated constant pH input file (cpin) by *cpinutil.py* program. Initially before production, we performed energy minimization, followed by heating and equilibrating the system at target temperature (300 K). To calculate predicted pK_a_ protonation states and population, the production MD was run at pH 5.0, 7.0, and 9.0. The Amber *sander* module was used for all types of MD. The analysis of MD results was done by program *cphstats* and *cpptraj* (38). The data obtained after analysis were plotted by *matplotlib*.

## Data availability

All data generated or analyzed during this study have been included in this article and its supplemental information. The IDs for the crystal structure PDB deposition are included in Table 1 and Table S1.

## Supporting information

This article contains supporting information (13, 18, 19, 39, 40).

## Conflict of interest

The authors declare that they have no conflicts of interest with the contents of this article.

## Supporting information

Supplemental Figures S1–S6, Tables S1–S2 and Note S1
