# Supplementary material for: Structural insights into the mechanism of pH-selective substrate specificity of the polysaccharide lyase Smlt1473
Source: J Biol Chem. 2021 Aug 3;297(4):101014. doi: 10.1016/j.jbc.2021.101014 (PMC8511899; doi:10.1016/j.jbc.2021.101014)
Supplement: Supplemental Figures S1–S6, Tables S1–S2 and Note S1 [file mmc1.docx]

Supplementary information

Text S1. Molecular Dynamic simulation of tetra-HA docked Smlt1473 structure

We analyzed superimposed structures of tetra-ManA co-crystallized and tetra-HA docked Smlt1473 structures, further rationalizing the differences in the mode of substrate binding as the reason behind substrate specific activity enhancing and diminishing catalytic tunnel mutants (see main text for further details). Validating the mode of substrate binding in the case of tetra-HA docked structure provides further evidence to support our conclusions. We performed MD simulations of 100 ns for tetra-HA docked Smlt1473 (see methods). The MD trajectory evaluated for the protein backbone r.m.s.d. and C_α_ r.m.s.f plots suggests the molecule is stable over the time course and the convergence of the simulation (reference structure: tetra-HA docked Smlt1473) (Figure S5A and Figure S5B). To visually depict the changes as a function of simulation time, we extracted the MD trajectory at 0, 20, 40, 60, 80, and 100 ns and superimposed it with tetra-HA docked Smlt1473 structure as the reference. We observed that, structural elements *viz.* N-terminal lid loop, helices and other loops which forms substrate entry and exit site showed significant fluctuations. Interestingly, the substrate position as well as the catalytic tunnel architecture is maintained throughout the simulation time-scale (Figure S5Ca-f). The substrate has shown maximal r.m.s.d. at the beginning (~0-30 ns) of simulation which is similar to what we observed for the protein backbone (Figure S5D). However, the r.m.s.d. is well within the limits of experimental error and shows insignificant deviation. Next, we superimposed the substrate trajectory in stick representation as function of time; 0 ns substrate trajectory was used as reference for the 20, 40, 60, 80, and 100 ns trajectory (Figure S5D). The substrate secondary structure superimposed well and confirms that its orientation and positioning is maintained throughout the simulation. We also analyzed the deviation of distances among three key catalytic interactions: (i) Y222’s hydroxyl O to tetra-HA C5 at +1 subsite, (ii) H168’s epsilon N (Nε) to tetra-Ha sugar ring’s O5 at +1 subsite, and (iii) N167’s delta N (Nδ) to carboxyl O6A at +1 subsite (Figure S5E). Except for (iii), the relative distances (i, ii) are maintained throughout the simulation time and account for the high probability to form H-bond (Figure S5Eb-h).

**Figure S1. Structural superimposition of Smlt1473 wildtype ‘apo’ crystal structure solved at different pH.** Structural superimposition in PyMol represented in cartoon form (A). Structural superimposition DONE by CCP4 program SUPERPOSE and is tabulated as r.m.s.d differences (Å) (B).

**Figure S2. Structural superimposition and analysis of substrate interacting residues of Smlt1473 wt ‘apo’ crystal structures crystalized at pH-5.0 (green), 7.0 (cyan), and 9.0 (magenta).** The substrate interacting residues (shown in stick representation) forming entry site (A.a), tunnel site (B.a), exit site (C.a). The corresponding substrate interacting residues 2F_o_-F_c_ electron density contour at sigma level 1.2 (A. b-d), (B. b-d), (C. b-d). The differences in rotamers are circled; there is no observable rotameric transition for the tunnel site.

**Figure S3.** **Docking of tetra-HA unit onto crystal structure of wildtype Smlt147 bound to tetra-ManA at pH 5.** Tetra-HA unit was taken from two sources (1) from PDB 1lxk (green) and (2) www.polysac3db.cermav.cnrs.fr (cyan) (A). The backbone of tetra-HA units superimposes well (0.86 A), with differences in the rotamers of substituents. This constitutes two ligand conformations. Rosetta flexible ligand protocol was used for docking. A total of 5000 models were generated for each ligand conformations. Here we have shown the details for docking of tetra-HA unit from source (1) (B). Rosetta energy score vs Interface delta score profile for all 5000 models (C). Interface delta score vs ligand r.m.s.d (Å) profile for top 20% models. Ligand r.m.s.d was calculated with respect to a native structure. The native structure was identified from the top 20% of models grouped based on energy score and then were sorted with respect to interface delta score. The native structure has the least interface delta score with reasonable ligand interactions with active site residues (D). Superposition of the native structures with tetra-HA from source (1) and (2). The binding modes are similar and no significant differences are observed.

**Figure S4. Conserved mode of binding in PL8 hyaluronate lyase from *S. pneumoniae*.** The substrate, hyaluronic acid (HA) enters from the +2 subsite and extend up to -4 subsite and is cleaved at -1, +1 subsite. HA is a polymer of a (1→3) linked sugar dimer unit BDP (BETA-D-GLUCOPYRANURONIC ACID) and NAG (N-ACETYL-D-GLUCOSAMINE), with each dimer unit constituting one HA unit. The arrangement of substrate in the PL cleft follows a trend in displaying faces of sugars in either **‘α’** (C1, C3 and, C5 are facing forward C2, C4 and, C6 are facing backward) or **‘β’** (C2, C4 and, C6 are facing forward and C1, C3 and, C5 are facing backward). In case of HA, it is important to note that sugars constituting one HA unit (BDP-NAG) always display the same face (either α or β), but the face of subsequent (1→4) linked HA unit changes from the preceding HA unit. This gives rise to alternate display of α and β faces. Interestingly in this case, where the same enzyme is co-crystallized with 3HA (1LOH), 2HA (1LXK) and, 1HA (1C82), the mode of HA binding is highly conserved at -2, -1 subsite as can be seen in superimposed crystal structures. In each case the -2, -1 subsite display a well superimposed β-face. By looking at 1LOH and 1LXK the +1, +2 unit seems to display specific mode of binding, but when we look at 1C82 crystal structure the other unlinked HA unit (not shown here) is oriented arbitrarily at the exit site corresponding to +1, +2 subsite and do not superimpose with +1, +2 of 1LOH and 1LXK. This demonstrates that HA binding at -2, -1 subsite is a major determinant of substrate orientation.

**Figure S5. Molecular dynamics simulation of tetra-HA docked Smlt1473 structure.** r.m.s.d. plot of protein backbone as a function of simulation time of 100 ns (A). Average C-α r.m.s.f plot of protein residues after MD (B). Structural superposition of MD trajectory extracted at 0, 20, 40, 60, 80, and 100 ns with tetra-HA docked Smlt1473 structure (C a-f). Substrate (tetra-HA or 4HA) r.m.s.d plot as a function of simulation time, stick representation of superposed substrate trajectory at 20, 40, 60, 80, and 100 ns (0 ns trajectory used as reference) (D). Deviation in distances of catalytically important residues as a function of simulation time (E a), tetra-HA docked Smlt1473’s catalytic geometry shown in stick representation (E b), catalytic geometry with same representation shown for trajectory extracted at 0, 20, 40, 60, 80, and 100 ns.

**Figure S6. Coulombic (Coul-SR: Protein-4HA) and Lennard-Jones (LJ-SR: Protein-4HA) potential interaction energy, Rosetta docking score energies derived from tetra-HA docked Smlt1473 structure MD and docking.** Interaction energy plot as a function of simulation time (A). Table listed for interaction and total interaction Smlt1473-tetraHA energy from MD and Rosetta docking (B).

**Table S1 X-ray data collection and refinement statistics**


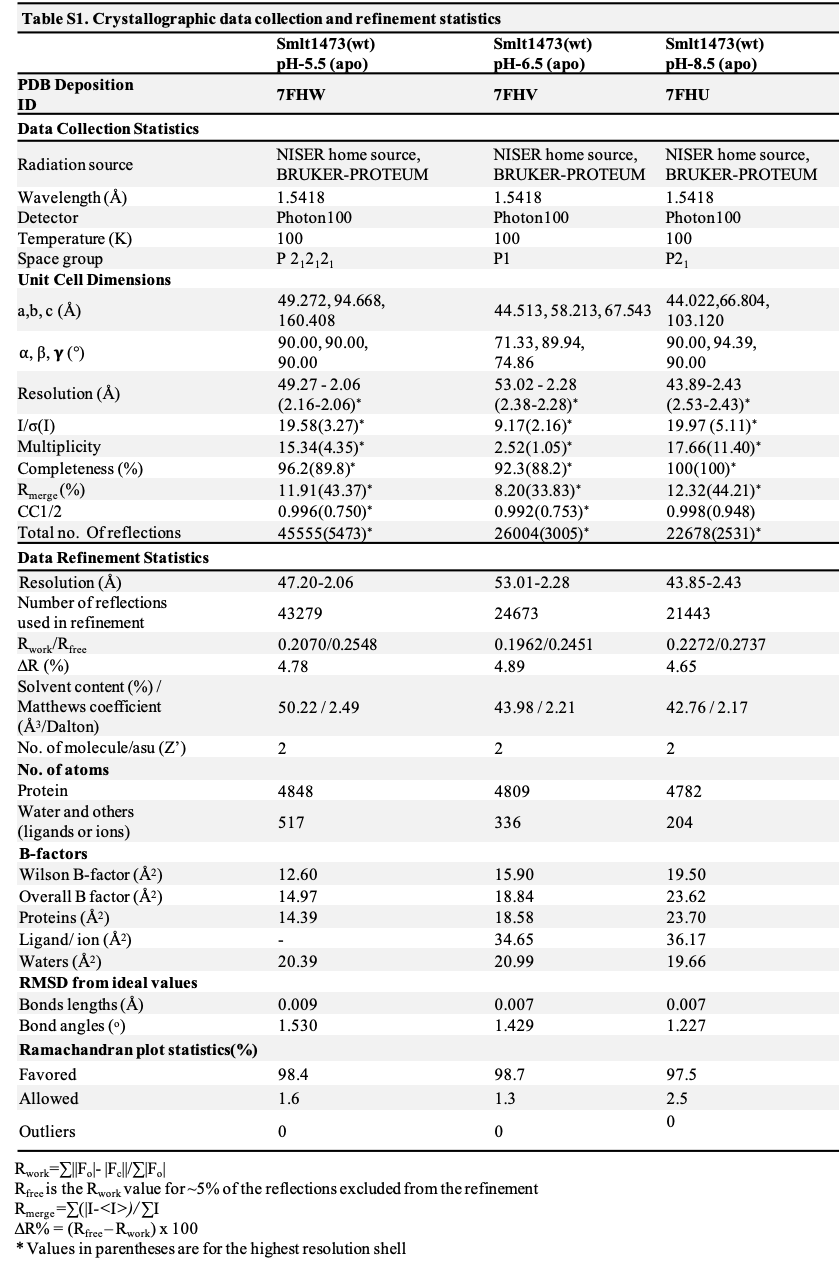


**Table S2 Optimum pH for monospecific PLs**

|  | | | | | | | |
| --- | --- | --- | --- | --- | --- | --- | --- |
| **PL family** | **Organism** | **PL type** | **EC No.** | **PDB ID (apo/substrate bound)** | **Optimum pH** | **Catalytic residues** | **Reference** |
| PL-8 | *Streptococcus pneumoniae* R6 | HA lyase | 4.2.2.1 | 1EGU (apo) / 1LOH (3HA bound) | 6 | HIS 399  TYR408 | 18 |
| PL-8 | *Streptococcus aglactiae* NEM316 | HA lyase | 4.2.2.1 | 1F1S (apo) / 1LXM (3HA bound) | 5 | H479  Y488 | 19 |
| PL-8 | *Streptomyces coelicolor* A3(2) | HA lyase | 4.2.2.1 | 2WCO (1HA bound) | 5.2 | H244  Y253 | 39 |
| PL-5 | *Sphingomonas* sp. A1 | polyManA lyase | 4.2.2.3 | 1QAZ (apo) / 4F1O (tetra-alginate) / 4F13 (tetra-alginate) | 8 | H192  Y246 | 13 |
| PL-5 | *Pseudomonas aeruginosa* PAO1 | polyManA lyase | 4.2.2.3 | 4OZW (apo) / 4OZV | 8.5 | H202  Y256 | <(https://www.rcsb.org/structure/4OZV>) |
| PL-7 | *Sphingomonas* sp. 1 | polyspecific alginate lyase | 4.2.2.- | 2CWS (apo) / 2ZAC (tetra-alginate) | 7.5 | H191  Y284 | 40 |


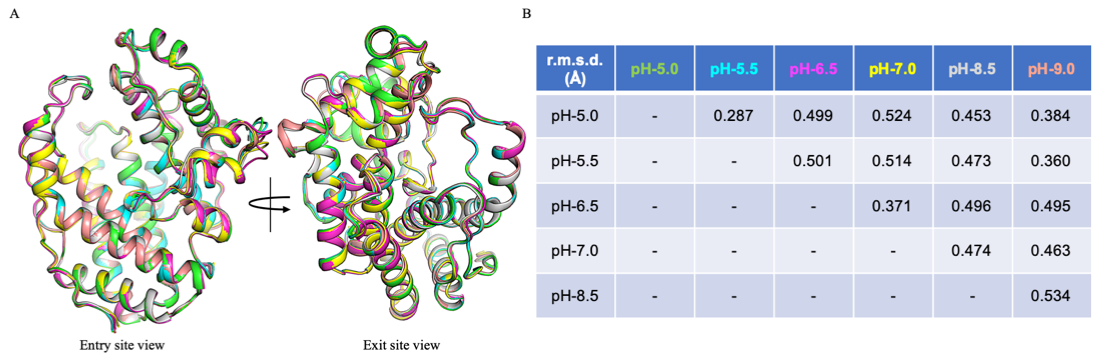

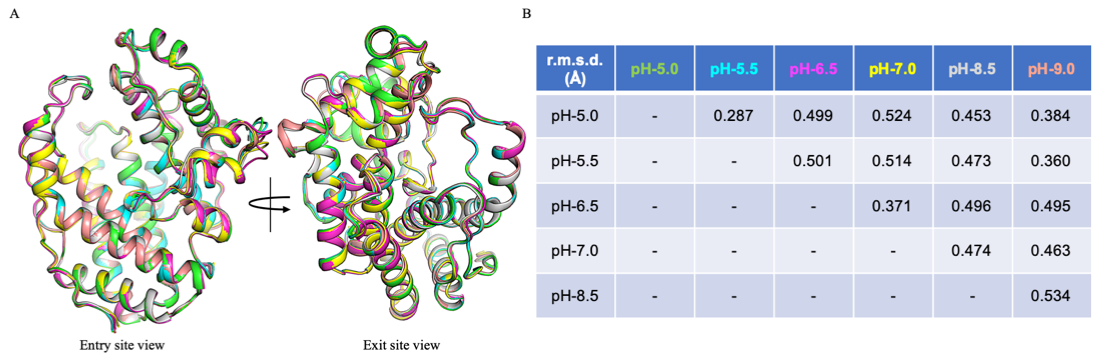


Entry site

view

Exit site

view

**A**

**B**

Figure S1


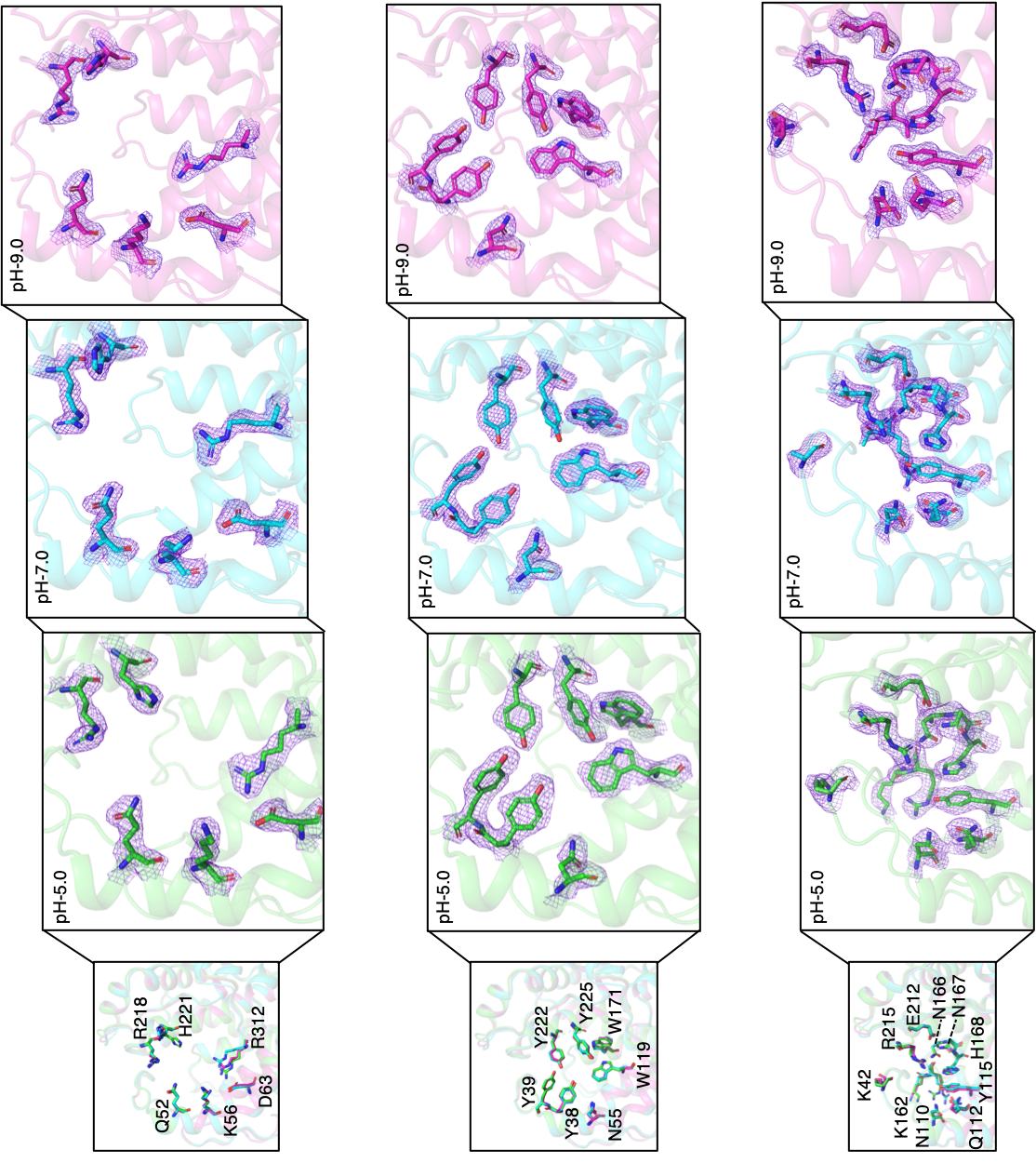


Figure S2

**A**

**B**

**C**

a

b

c

d

a

b

c

d

a

b

c

d


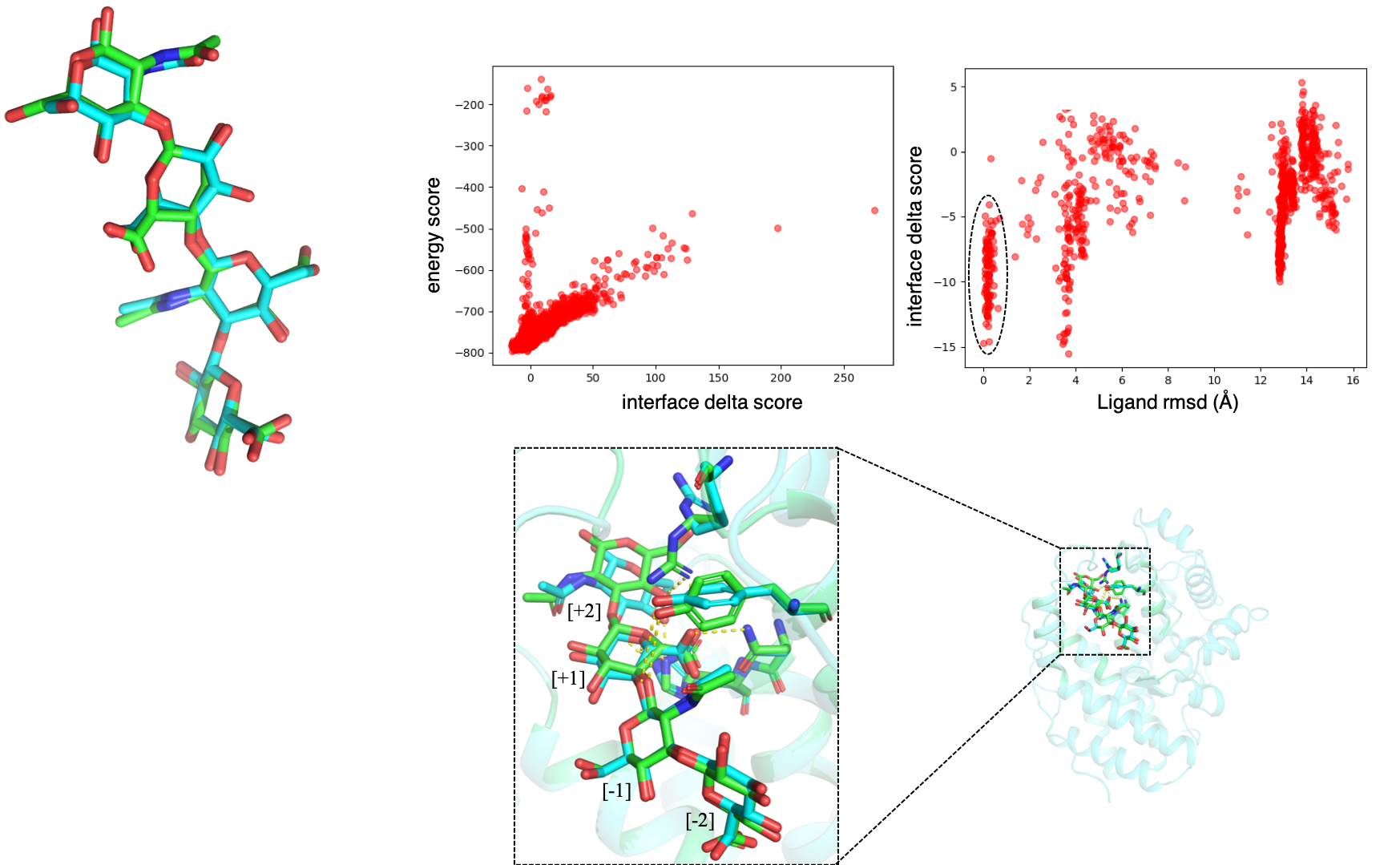


**B**

**C**

**D**

**A**

Figure S3


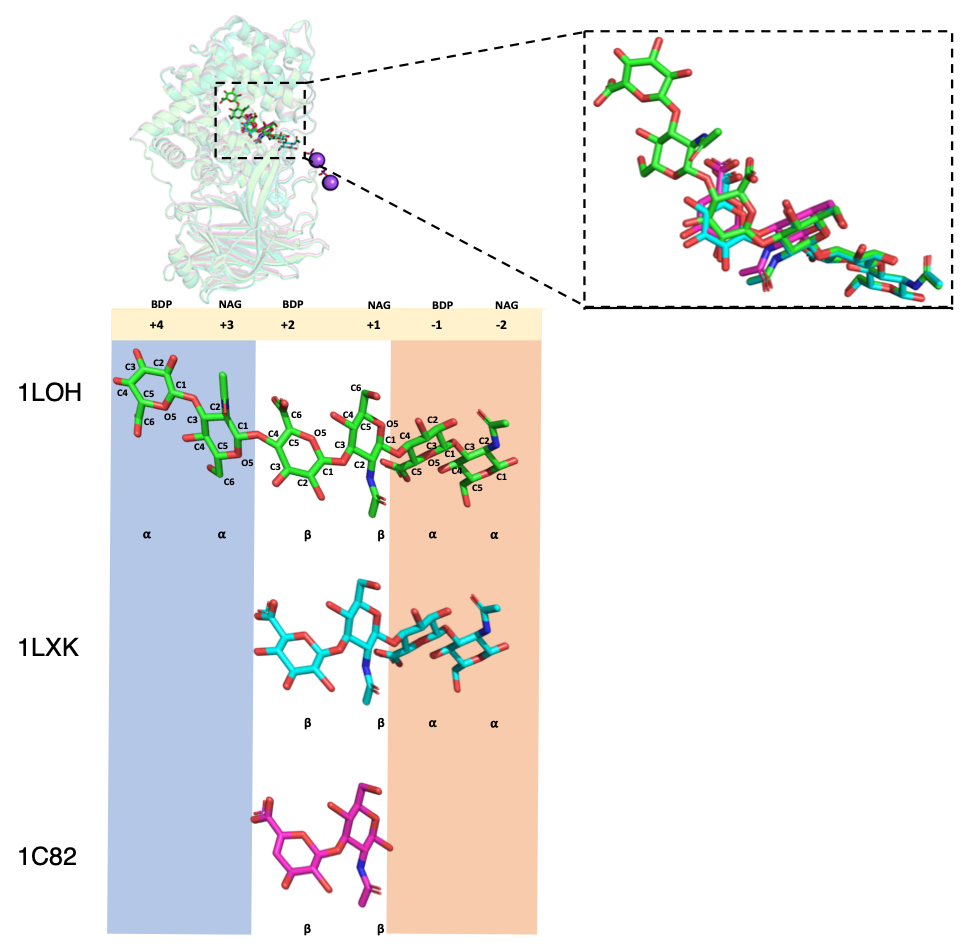


Figure S4


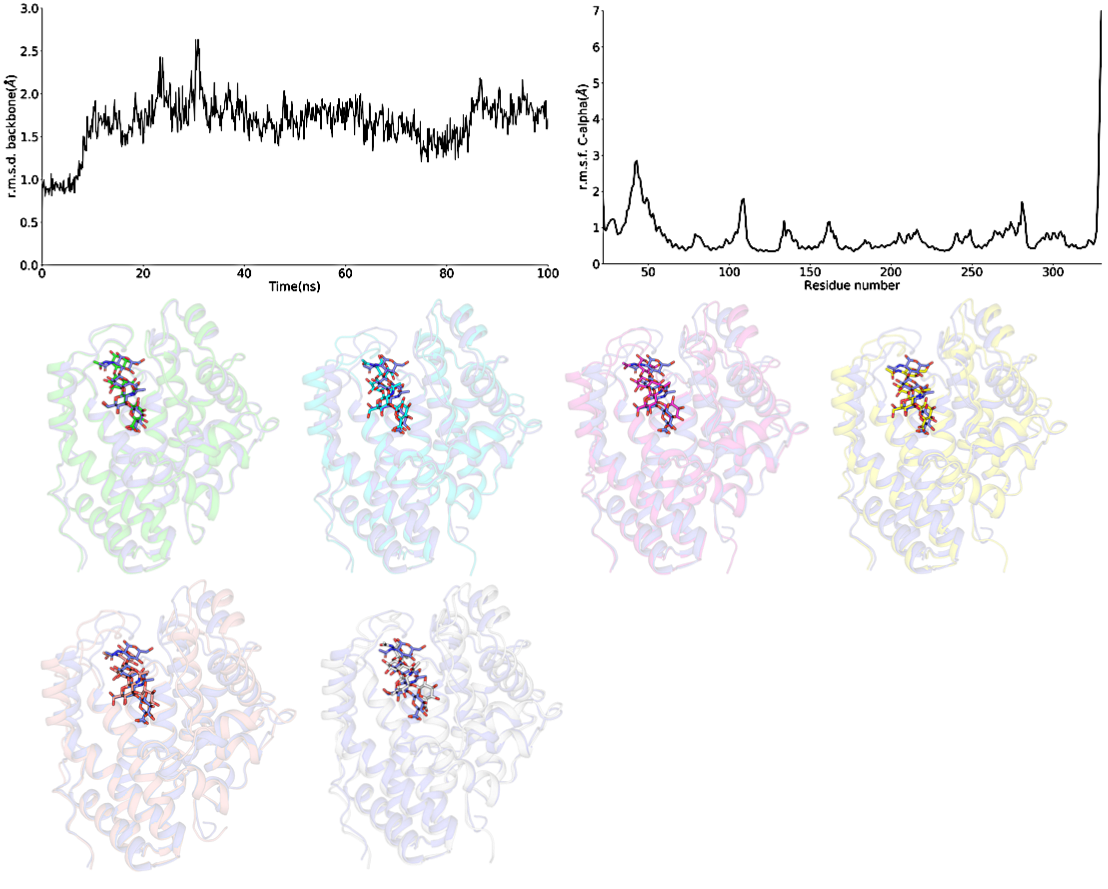

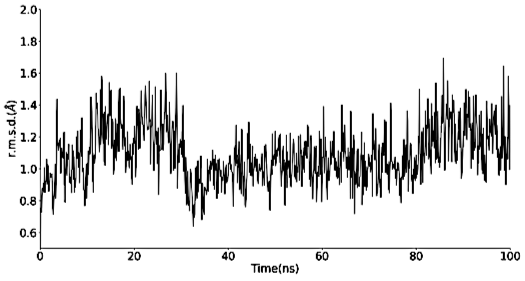

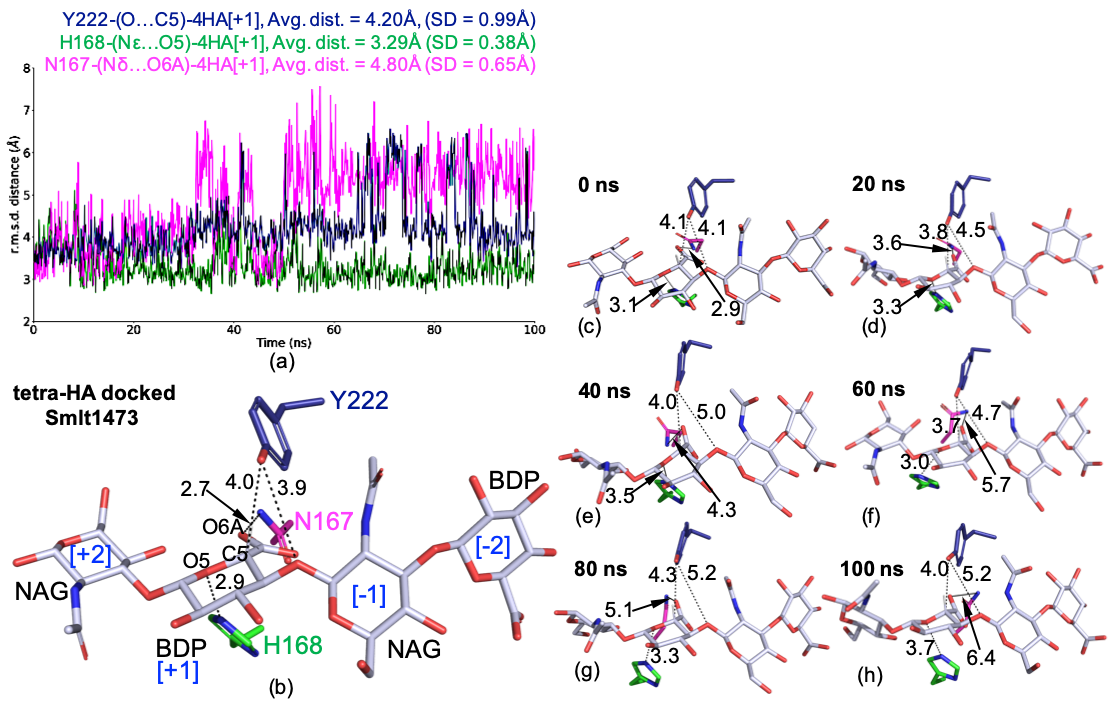


0🡪40ns

0.963Å

0🡪60ns

1.00Å

0🡪80ns

1.08Å

0🡪100ns

1.4Å

0 ns

20 ns

40 ns

60 ns

80 ns

100 ns


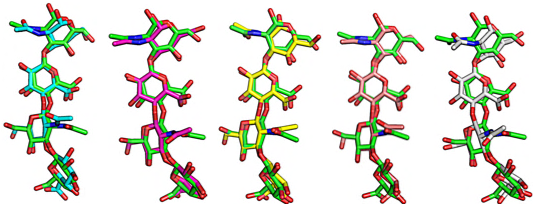


0🡪20ns

1.16Å

**A**

**B**

**C**

**D**

**E**

(a)

(b)

(c)

(d)

(e)

(f)

Figure S5


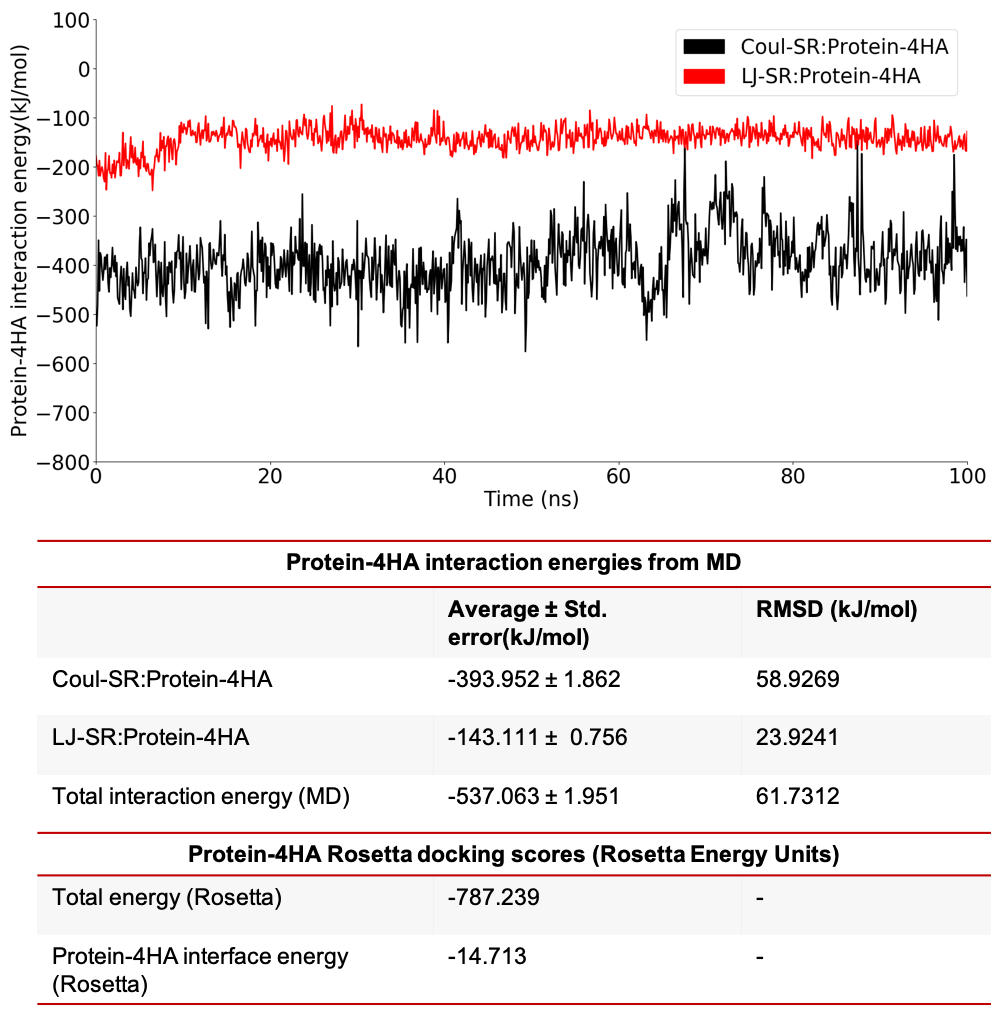


Figure S6
